# Zebrafish as a model for apolipoprotein biology: comprehensive expression analysis and a role for ApoA-IV in regulating food intake

**DOI:** 10.1242/dmm.018754

**Published:** 2015-01-29

**Authors:** Jessica P. Otis, Erin M. Zeituni, James H. Thierer, Jennifer L. Anderson, Alexandria C. Brown, Erica D. Boehm, Derek M. Cerchione, Alexis M. Ceasrine, Inbal Avraham-Davidi, Hanoch Tempelhof, Karina Yaniv, Steven A. Farber

**Affiliations:** 1 Carnegie Institution for Science, Department of Embryology, Baltimore, MD 21218, USA; 2 Johns Hopkins University, Department of Biology, Baltimore, MD 21218, USA; 3 Weizmann Institute of Science, Department of Biological Regulation, Rehovot 7610001, Israel

**Keywords:** Zebrafish, Developmental expression patterns, mRNA *in situ* hybridization, Apolipoprotein A-I, Apolipoprotein B, Apolipoprotein A-IV, Apolipoprotein E, Regulation of food intake

## Abstract

Improved understanding of lipoproteins, particles that transport lipids throughout the circulation, is vital to developing new treatments for the dyslipidemias associated with metabolic syndrome. Apolipoproteins are a key component of lipoproteins. Apolipoproteins are proteins that structure lipoproteins and regulate lipid metabolism through control of cellular lipid exchange. Constraints of cell culture and mouse models mean that there is a need for a complementary model that can replicate the complex *in vivo* milieu that regulates apolipoprotein and lipoprotein biology. Here, we further establish the utility of the genetically tractable and optically clear larval zebrafish as a model of apolipoprotein biology. Gene ancestry analyses were implemented to determine the closest human orthologs of the zebrafish apolipoprotein A-I (*apoA-I*), *apoB*, *apoE* and *apoA-IV* genes and therefore ensure that they have been correctly named. Their expression patterns throughout development were also analyzed, by whole-mount mRNA *in situ* hybridization (ISH). The ISH results emphasized the importance of apolipoproteins in transporting yolk and dietary lipids: mRNA expression of all apolipoproteins was observed in the yolk syncytial layer, and intestinal and liver expression was observed from 4–6 days post-fertilization (dpf). Furthermore, real-time PCR confirmed that transcription of three of the four zebrafish *apoA-IV* genes was increased 4 hours after the onset of a 1-hour high-fat feed. Therefore, we tested the hypothesis that zebrafish ApoA-IV performs a conserved role to that in rat in the regulation of food intake by transiently overexpressing ApoA-IVb.1 in transgenic larvae and quantifying ingestion of co-fed fluorescently labeled fatty acid during a high-fat meal as an indicator of food intake. Indeed, ApoA-IVb.1 overexpression decreased food intake by approximately one-third. This study comprehensively describes the expression and function of eleven zebrafish apolipoproteins and serves as a springboard for future investigations to elucidate their roles in development and disease in the larval zebrafish model.

## INTRODUCTION

The current epidemic of metabolic syndrome has resulted in widespread morbidity and mortality due to population increases in obesity, cardiovascular disease, type II diabetes, hypertension and stroke (Grundy et al., 2005). Developing treatments for the dyslipidemias frequently associated with metabolic syndrome [e.g. high serum triglycerides and low high-density lipoprotein (HDL) cholesterol] is a priority. It is well known that lipoprotein particles transport lipids throughout the circulation. However, the development of improved treatments for common dyslipidemias requires a deeper understanding of lipoprotein function and regulation. For instance, despite a vast scientific literature spanning over 50 years, we still lack a basic mechanistic understanding of the relationship between HDL and cardiovascular disease risk (Voight et al., 2012). Furthermore, characterization of lipoprotein biology is impeded without a comprehensive study of apolipoproteins, the class of over a dozen secreted, lipid-binding proteins that function as structural backbones of lipoprotein particles and regulators of cellular lipid flux through their interactions with cell surface receptors.

This study focuses on four major serum apolipoproteins: apolipoprotein A-I (APOA-I), apolipoprotein B (APOB), apolipoprotein E (APOE) and apolipoprotein A-IV (APOA-IV) (note that we use capitals to denote the mammalian protein form). APOA-I is the main protein component of HDL particles. In addition to its intrinsic, beneficial anti-oxidative, anti-inflammatory and anti-bacterial properties (Feingold and Grunfeld, 2011; Garner et al., 1998; Navab et al., 2000a; Navab et al., 2000b), APOA-I regulates cholesterol efflux from cells to HDL particles, the first step in transporting peripheral cholesterol to the liver for excretion in bile. The liver and intestine produce mammalian APOA-I and subsequently HDL. Humans have two isoforms of the second apolipoprotein of interest, APOB, resulting from mRNA editing: the short form APOB-48 is produced by the intestine, and the long form APOB-100 by the liver (Fisher and Ginsberg, 2002). APOB-48 is an essential structural component of intestinally secreted chylomicrons, whereas APOB-100 gives structure to very-low-density lipoproteins (VLDL) and low-density lipoproteins (LDL) of hepatic origin. APOE, the third apolipoprotein of interest, is exchangeable, transferring between most classes of lipoproteins in the circulation. APOE binds to LDL receptors, thereby regulating lipid uptake (Mahley, 1988). APOE-deficient mice develop severe atherosclerosis due to increased circulating LDL cholesterol (Nakashima et al., 1994). In addition to being expressed by the intestine, liver and macrophages, APOE is synthesized in the mammalian brain and acts as the major apolipoprotein of the lipid-rich central nervous system (CNS). The final apolipoprotein examined, APOA-IV, is produced by the intestine and secreted as a component of chylomicrons following lipid-rich meals. In rats, acute administration of APOA-IV suppresses food intake (Fujimoto et al., 1992; Fujimoto et al., 1993b; Wang et al., 2013a).

**RESOURCE IMPACT**

**Background**

The numerous diseases related to metabolic syndrome – such as obesity, cardiovascular disease, type II diabetes, hypertension and stroke – are often associated with severe morbidity and mortality. An improved understanding of lipoproteins, which transport lipids throughout the circulation, is vital for developing new pharmaceutical treatments for lipid abnormalities associated with metabolic syndrome. Greater knowledge of apolipoproteins (proteins that bind lipids and that function both as structural backbones of lipoprotein particles and as regulators of cellular lipid import and export) will prove to be invaluable to our understanding of lipoprotein function. Mouse and cell culture models are often used to study dyslipidemias (abnormal levels of lipids in the blood) and apolipoprotein biology. However, constrains of these systems warrant development of a new model that can replicate the complex *in vivo* milieu that regulates lipoproteins biology.

**Results**

In this investigation, the authors took advantage of the highly genetically tractable and optically accessible larval zebrafish as a model to investigate apolipoprotein dynamics and actions. The spatiotemporal mRNA expression of the 11 zebrafish apolipoprotein genes (*apoA-I*, *apoB*, *apoE* and *apoA-IV*) was characterized from embryogenesis through early larval development. All apolipoproteins were expressed in the yolk syncytial layer, highlighting their importance in transporting lipids from the yolk cell to the developing embryo, and subsequently in the digestive organs (intestine and liver). Given that apolipoproteins are essential for lipoprotein formation and dietary lipid transport, their regulation by a high-fat diet was investigated. mRNA expression of three out of the four *apoA-IV* paralogs was increased by a high-fat feed. It was also shown, by acutely overexpressing ApoA-IVb.1 in transgenic zebrafish and observing a decrease in the consumption of fluorescently labeled lipids, that zebrafish ApoA-IV has a role in the regulation of food intake similar to that of rat APOA-IV.

**Implications and future directions**

This model lays the foundation for future studies on apolipoproteins and their associated lipoproteins and dyslipidemias in zebrafish. The discoveries presented here emphasize the close relationship between zebrafish and human apolipoprotein expression, further establishing the zebrafish as a novel model organism for the study of apolipoprotein biology. These findings encourage researchers to apply the unique attributes of the larval zebrafish (rapid development, genetic tractability, live imaging, visualization of fluorescently labeled lipids, and ease of large pharmaceutical screens in live animals) to the study of global and subcellular apolipoprotein dynamics in development, health and disease.

Our choice to focus on these four apolipoproteins in this investigation was determined by their implication in a diverse range of dyslipidemias and neurodegenerative diseases. For example, there are striking positive correlations in human populations between protection from cardiovascular disease and the levels of APOA-I and HDL cholesterol, as well as the occurrence of specific APOA-I polymorphisms (Franceschini et al., 1980; Kannel et al., 1964; Toth et al., 2013). By contrast, LDL cholesterol levels, which are in part dependent on APOB owing to its required structural role in these particles, are positively associated with cardiovascular disease risk (Wilson et al., 1998). Three polymorphisms in human APOE are strongly associated with the efficacy of plasma lipid clearance, and consequently with the risk of cardiovascular disease; these alleles are also correlated with Alzheimer’s disease risk (Corder et al., 1993; Utermann et al., 1977). Finally, the action of APOA-IV in reducing food intake is reduced upon chronic high-fat feeding and obesity, potentially furthering the cycle of hyperphagia and weight gain (Tso and Liu, 2004).

Cell culture and mouse studies have provided the bulk of our current understanding of the mechanistic relationships between apolipoproteins and disease. However, cell culture systems do not replicate the crosstalk between multiple organs, and studies performed in whole animals often have to rely on indirect assays that measure surrogates for lipid metabolism (e.g. plasma lipid profile). As a result, the details of the regulatory signals that coordinate cellular responses to lipids and production of specific lipoproteins from particular tissues are not well characterized. For example, it was long believed that APOA-I and HDL prevented cardiovascular disease through reverse cholesterol transport, the transport of peripheral cholesterol to the liver; however, mice lacking APOA-I have normal biliary cholesterol secretion (Amigo et al., 2011) and some pharmaceutical agents that raise HDL-C fail to provide the protection observed with naturally high HDL cholesterol levels (Barter et al., 2007; Boden et al., 2011). Moreover, acute administration of APOA-IV to rats suppresses food intake (Fujimoto et al., 1993b), but chronic overexpression or deletion of APOA-IV does not affect food intake in mice (Aalto-Setälä et al., 1994; Weinstock et al., 1997). The seemingly conflicting findings surrounding how APOA-I and APOA-IV function highlight the need for experimental model systems that support the study of global apolipoprotein dynamics in living organisms and relate these observations back to the response of a particular cell within a living organ. Remarkably, studies at this level of resolution can be performed in the larval zebrafish (*Danio rerio*).

The larval zebrafish has a highly similar, yet simplified, gastrointestinal tract to that of humans (Carten and Farber, 2009). Zebrafish also have a similar lipoprotein lipid transport system (Babin and Vernier, 1989), although the teleost genome duplication event created multiple paralogs of most zebrafish apolipoproteins. Unlike humans, but similar to mice, zebrafish transport greater amounts of plasma cholesterol as HDL than as LDL (Pedroso et al., 2012). Historically an important developmental model, the zebrafish is emerging as an invaluable resource to study metabolic function in health and disease (Anderson et al., 2011; Asaoka et al., 2013; Carten and Farber, 2009; Fang and Miller, 2012; Otis and Farber, 2013; Schlegel and Stainier, 2007; Seth et al., 2013). This claim is supported by a body of research that has combined the genetic tractability and conduciveness to live imaging of larvae with novel zebrafish models of dietary manipulations [high-fat (Carten et al., 2011; Marza et al., 2005), high-cholesterol (Stoletov et al., 2009) and high-carbohydrate diets (Fang et al., 2014; Wang et al., 2013b)], cardiovascular disease (Fang et al., 2011), type II diabetes (Curado et al., 2007; Pisharath et al., 2007), hepatic steatosis (Matthews et al., 2009; Passeri et al., 2009; Sadler et al., 2005) and obesity (Chu et al., 2012; Oka et al., 2010; Song and Cone, 2007), to provide translational insights that can be applied to human metabolic disorders.

Specific discoveries related to apolipoprotein function that have been gained from work in larval zebrafish include the findings that ApoB negatively regulates angiogenesis through Vegfr1 (Avraham-Davidi et al., 2012), ApoA-I binding protein inhibits angiogenesis through Vegfr2 (Fang et al., 2013), and ApoA-II is required for proper chromosome separation during nuclear division of *in vivo* larval zebrafish cells and cultured human cells (Zhang et al., 2011). Although multiple studies have described expression patterns and functions of particular zebrafish apolipoproteins (Babin et al., 1997; Herbomel et al., 2001; Monnot et al., 1999; Poupard et al., 2000; Thisse et al., 2001; Thisse and Thisse, 2005; Tingaud-Sequeira et al., 2006), none have characterized expression from the eight-cell stage comprehensively throughout larval development, included all paralogs of a given gene or examined the effect of feeding.

For this reason we undertook a study to further establish the use of the zebrafish as a model of apolipoprotein function in lipid metabolism, embryonic development, dietary nutrient processing and disease. We first validated and clarified the zebrafish apolipoprotein nomenclature by investigating the ancestral relationships between the four human and 11 zebrafish genes encoding the APOA-I, APOB, APOE and APOA-IV proteins by performing sequence, syntenic, and phylogenetic analyses. Second, we characterized zebrafish apolipoprotein spatiotemporal expression patterns throughout embryogenesis and early larval development, and determined how these metabolic proteins are transcriptionally regulated by a high-fat feed. Finally, we determined that zebrafish ApoA-IV, of which three of four paralogs are upregulated by a high-fat meal, decreases food intake when overexpressed in transgenic larvae presented with a high-fat meal. These results indicate that zebrafish ApoA-IV has a conserved role in the regulation of high-fat food intake and further establishes the larval zebrafish as a valuable model of apolipoprotein biology.

## RESULTS

### Gene ancestry analysis of zebrafish apolipoproteins

The application of a common apolipoprotein nomenclature is of paramount importance to the larger research community in order to correctly interpret, replicate and build upon published findings. By convention, zebrafish genes are named based on their closest human orthologs, as determined by predicted amino acid sequence comparisons. To verify that each zebrafish apolipoprotein gene was correctly named, we checked not only that the 11 zebrafish *apoA-I*, *apoB*, *apoE* and *apoA-IV* genes were most similar in amino acid sequence to their human ortholog, but also investigated shared syntenic gene regions and completed phylogenetic analyses. These data were communicated to the Zebrafish Model Organism Database (ZFIN) team to refine the nomenclature and propagate these gene names to zebrafish genome databases.

**Fig. 1.**
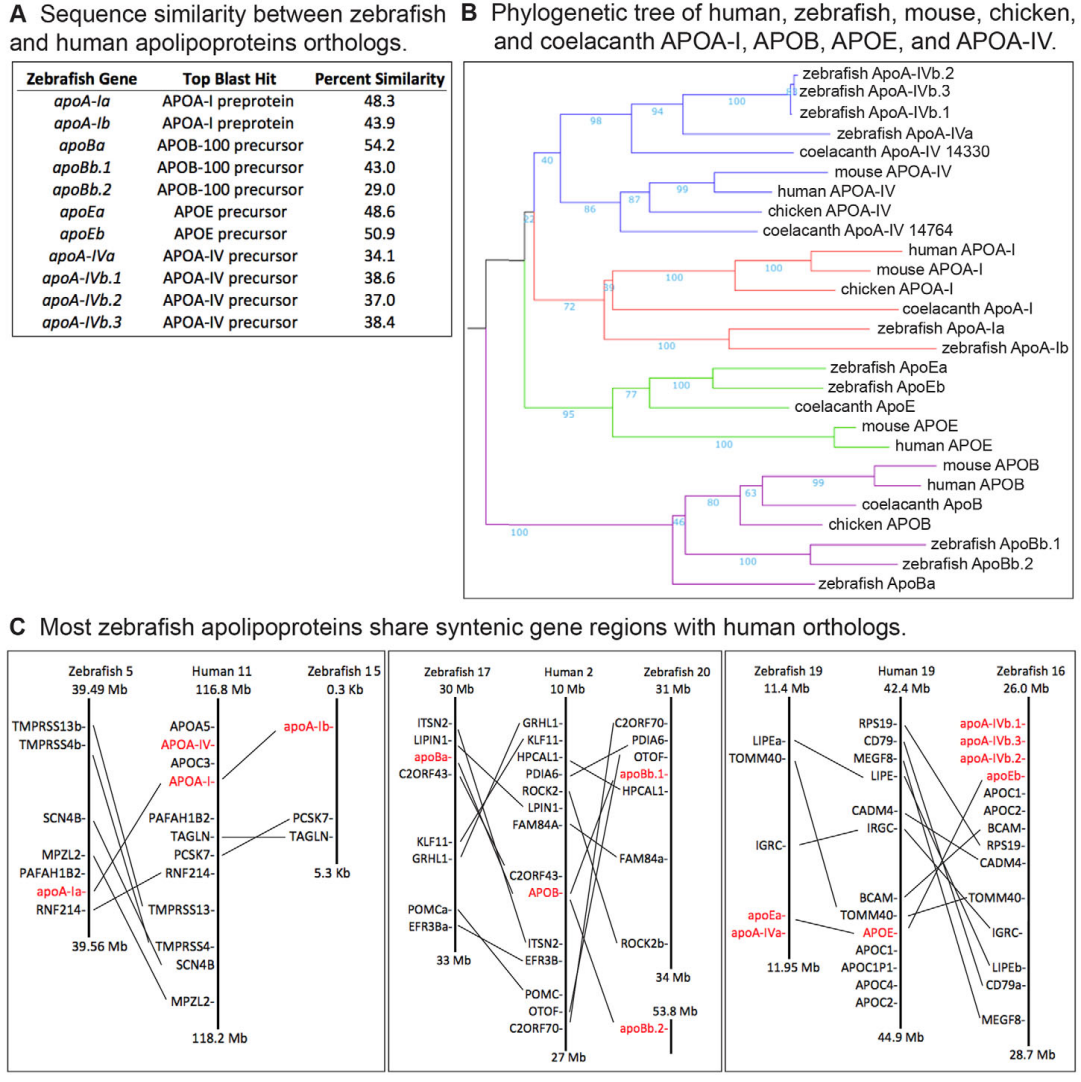
**Apolipoprotein gene ancestry analysis.** (A) There is low sequence similarity between zebrafish and human apolipoprotein orthologs. Zebrafish genes are listed next to the top BLASTp hit against the human RefSeq protein database. The percentage similarity between zebrafish and human orthologs was determined by Needleman–Wunsch amino acid pairwise sequence alignment. (B) Phylogentetic tree of human, zebrafish, mouse, chicken and coelacanth APOA-I, APOB, APOE and APOA-IV assembled from amino acid sequences as described in the Materials and Methods. Bootstrap support for each clade is reported in light blue (≥70 interpreted as significant). (C) Most zebrafish apolipoprotein genes share syntenic gene regions with their human orthologs. Schematic representation of human and zebrafish chromosomes regions with lines connecting human and zebrafish orthologs.

The gene ancestry analysis began with a comparison of zebrafish apolipoprotein amino acid sequences to the human reference protein sequence database using the BLASTp algorithm to verify orthology (Fig. 1A). This was followed by pairwise Needleman–Wunsch protein sequence alignments, which showed a range of 17.9–33.3% sequence identity and 29.0–54.2% sequence similarity between zebrafish and human orthologs (Fig. 1A; supplementary material Fig. S1).

The phylogenetic relationships between the human (*Homo sapiens*), zebrafish, mouse (*Mus musculus*), chicken (*Gallus gallus*) and coelacanth (*Latimeria chalumnae*) genes encoding the APOA-I, APOB, APOE and APOA-IV proteins were examined by generating a multiple sequence alignment with the iterative MAFFT multiple alignment tool. The resulting alignment was funneled into the MAFFT tree server and used to build a neighbor-joining tree with bootstrap resampling. The zebrafish genes encoding ApoA-I, ApoE and ApoB fell into well-supported clades with their vertebrate orthologs (Fig. 1B). In contrast, the zebrafish ApoA-IV paralogs grouped into a distinct subfamily of ApoA-IV genes that is only tenuously linked to amniote APOA-IV genes. The analysis also emphasizes that ApoA-IVb.1, ApoA-IVb.2 and ApoA-IVb.3 are evolutionarily more closely related to each other than to ApoA-IVa, as evidenced by very short branch lengths between the three ApoA-IVb paralogs (Fig. 1B).

The publicly available Genomicus and Synteny DB analysis tools were used to identify syntenic blocks of gene regions shared between zebrafish and human apolipoprotein orthologs. The syntenic analysis revealed that the human and zebrafish *APOA-I* and *APOE* orthologs clearly share syntenic gene regions (Fig. 1C). *apoBa* and *apoBb.1* also share syntenic gene regions with human *APOB*, but *apoBb.2* does not (Fig. 1C). The zebrafish *apoA-IV* genes share syntenic gene regions with human *APOE* (Fig. 1C).

### Spatiotemporal apolipoprotein expression patterns

Utilization of the zebrafish to model dyslipidemias is limited by incomplete knowledge of the developmental expression patterns of each apolipoprotein. Although expression patterns for a subset of zebrafish apolipoproteins have been published, these studies generally do not indicate which apolipoprotein paralog was studied, nor comprehensively describe where and when the genes are expressed throughout embryonic and larval development. Thus, we performed a comprehensive mRNA expression analysis of all 11 of the *apoA-I*, *apoB*, *apoE* and *apoA-IV* genes from the eight-cell stage to 6 days post-fertilization (dpf).

**Fig. 2.**
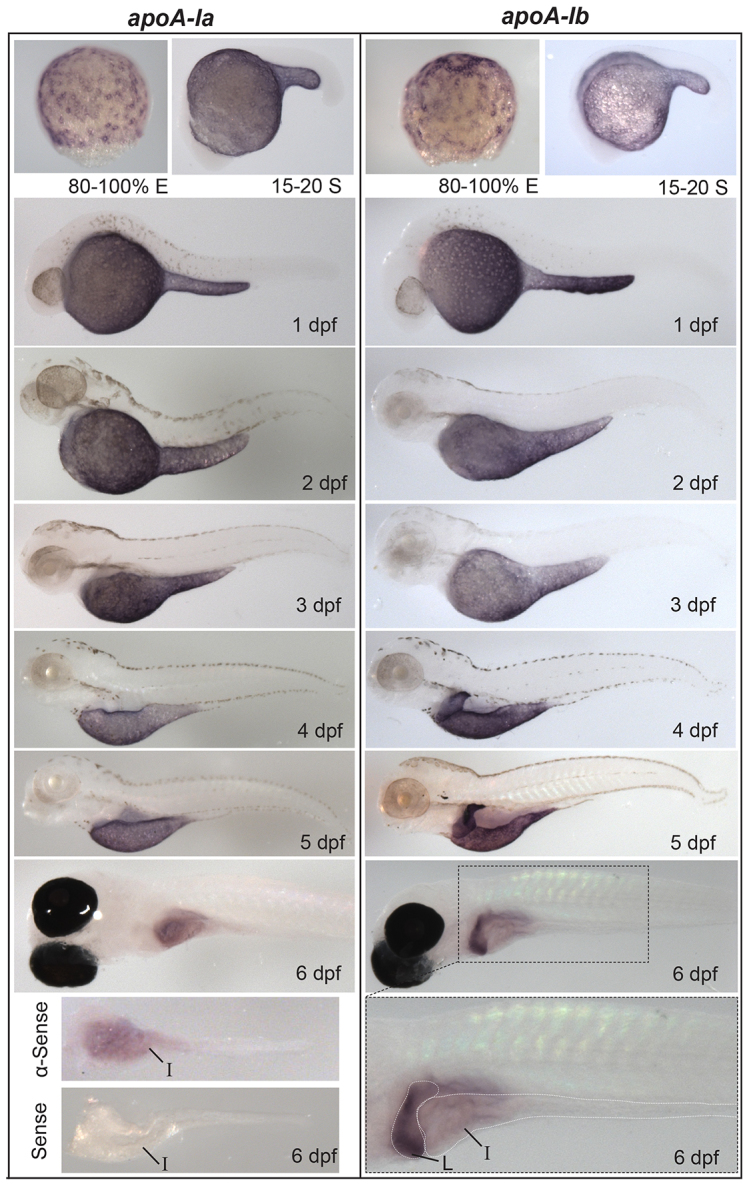
**Developmental mRNA expression patterns of *apoA-Ia* and *apoA-Ib*.** *In situ* hybridization of *apoA-Ia* and *apoA-Ib* during gastrulation [80–100% epiboly (E)], somitogenesis [15–20 somite (S)], and daily until 6 dpf. Both genes localize to the YSL from somitogenesis through 5 dpf, *apoA-Ia* localizes to the intestine (I) at 6 dpf, and *apoA-Ib* localizes strongly to the liver (L) and weakly to the intestine at 6 dpf. All zebrafish are wild type except 6-dpf larvae, which are nacre^−/−^. Larvae studied at 2–5 dpf were treated with PTU to prevent pigment formation. No signal was observed for either gene at the eight-cell stage or blastulation (supplementary material Fig. S1). Experiments were performed three times for each gene at each stage with *n*≥5 embryos or larvae per probe per experiment.

**Fig. 3.**
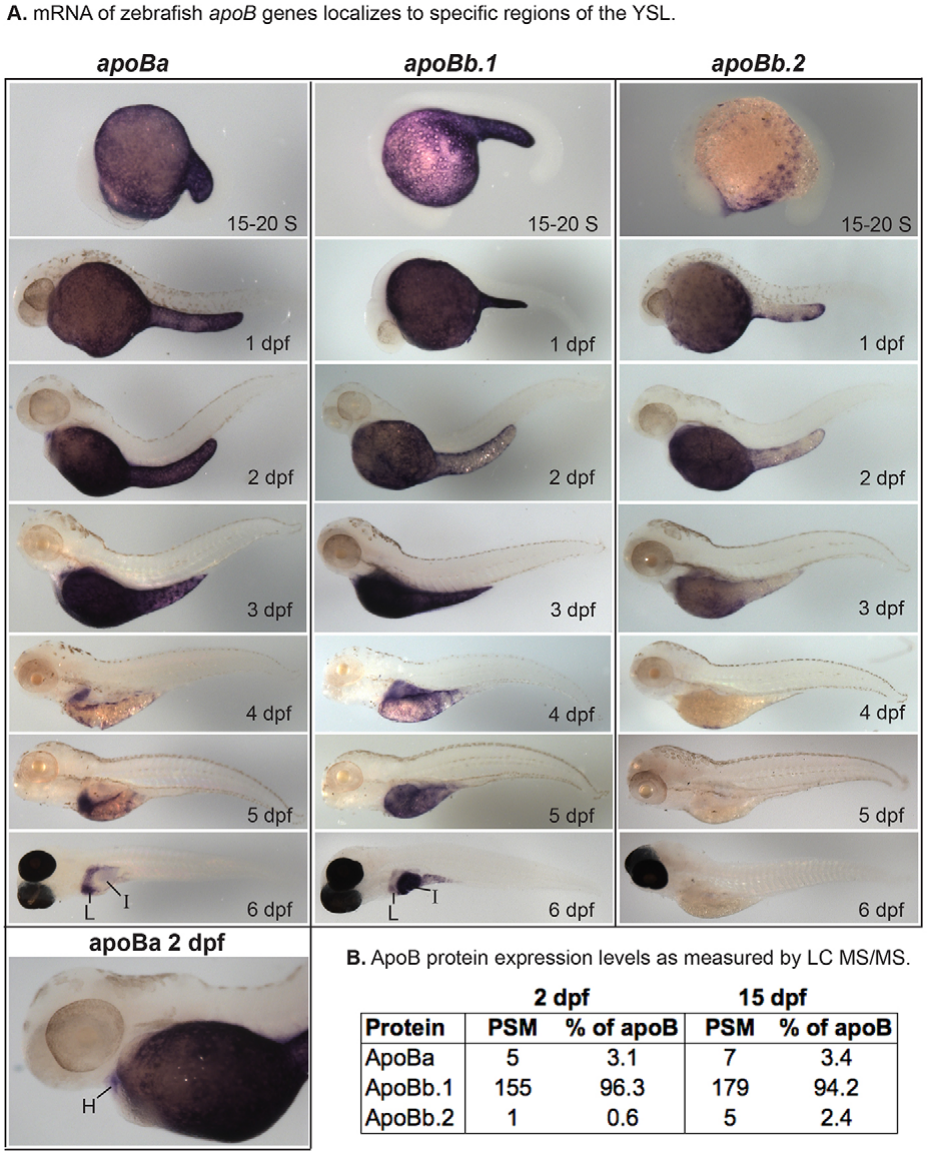
**Zebrafish *apoB* mRNA and protein expression.** (A) Localization of *apoBa*, *apoBb.1* and *apoBb.2* mRNA by ISH from somitogenesis [15–20 somite (S)] to 6 dpf. All *apoB* genes are expressed in the yolk syncytial layer (YSL), but mRNAs localize to distinct subregions of the YSL at various developmental stages. *apoBa* mRNA is present in the heart (H) at 2 dpf. At 6 dpf *apoBa* mRNA is expressed strongly in the liver (L) and weakly in the intestine (I), *apoBb.1* is strongly expressed in the intestine and weakly in the liver, and *apoBb.2* is not detectable by ISH. All zebrafish are wild type except 6-dpf larvae which are nacre^−/−^. Larvae collected at 2–5 dpf were treated with PTU to prevent pigment formation. No signal was observed at the eight-cell stage or at 30% or 80–100% epiboly (supplementary material Fig. S2). Experiments were performed three times for each gene at each stage with *n*≥5 embryos or larvae per probe per experiment. (B) Relative abundance of zebrafish ApoB paralogs as identified by peptide spectrum matches (PSM) in 2- and 15-dpf larvae (20–30 pooled larvae per experiment) as determined by LC-MS/MS of total larval proteins ≥250 kDa. Two experiments were performed for each developmental stage and both experiments yielded similar results. Data presented are from one experiment.

**Fig. 4.**
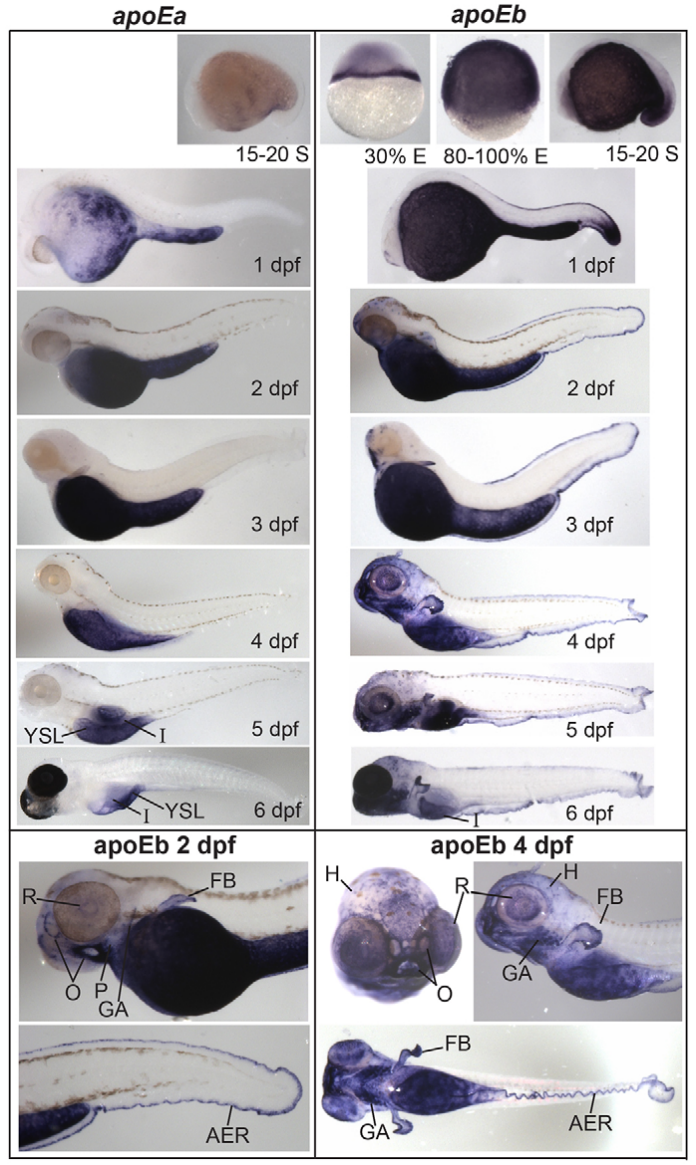
***apoEa* localization to the YSL and intestine contrasts with widespread expression of *apoEb*.** *apoEa* and a*poEb* mRNA expression observed by ISH during blastulation [30% epiboly (E)], gastrulation (80% E), somitogenesis [15–20 somite (S)] and 1–6 dpf. *apoEa* mRNA is expressed in distinct subregions of the YSL at somitogenesis and 1 dpf, and ubiquitously throughout the YSL from 2–5 dpf. At 6 dpf (but not 5 dpf), *apoEa* is observed in the intestine (I). *apoEb* is expressed not only throughout the YSL (30% E to 5 dpf), but also in the tail bud (15–20 S and 1 dpf), the apical ectodermal ridge (AER) (1–6 dpf), the head (H) (1–6 dpf), mouth and nose orifices (O) (2–6 dpf), the find buds (FB) (2–6 dpf), pharynx (P) (2–6 dpf), gill arches (GA) (3–6 dpf), periderm (4–6 dpf), retina (R) (4–6 dpf), intestine (6 dpf) and swim bladder (6 dpf). No expression of *apoEa* was observed during blastulation or gastrulation, or for either gene at the eight-cell stage (supplementary material Fig. S3). Zebrafish are wild type, except for 6-dpf larvae which are nacre^−/−^; 1–6 dpf larvae were treated with hydrogen peroxide to remove pigmentation. ISH was performed in triplicate with *n*≥5 embryos or larvae per probe per experiment.

**Fig. 5.**
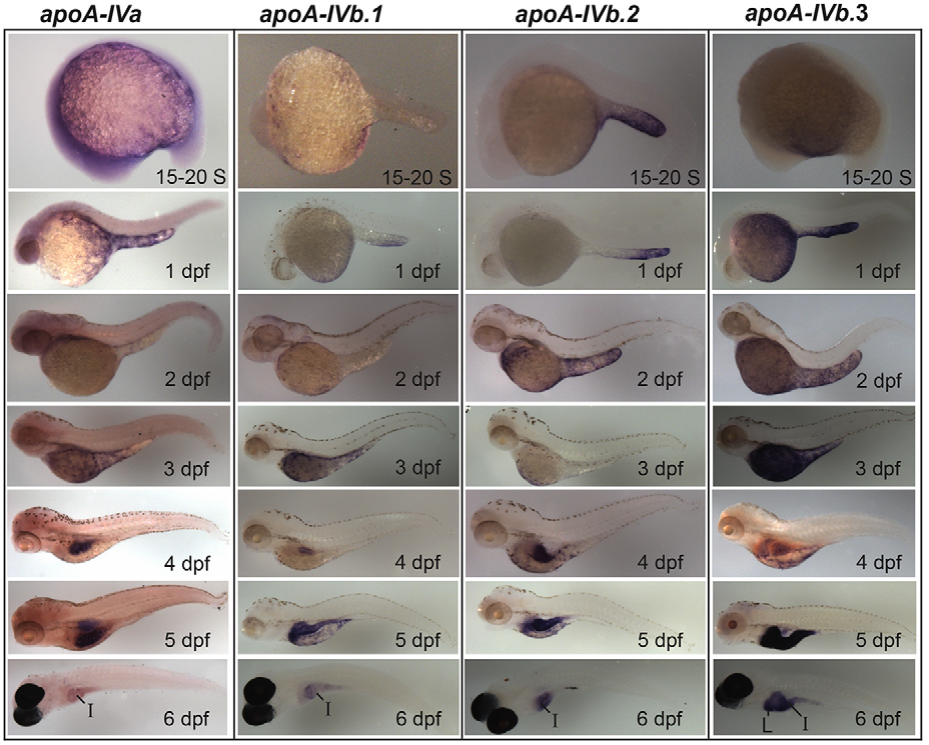
**The zebrafish *apoA-IV* genes have unique expression patterns in the YSL, intestine and liver.** Developmental mRNA expression dynamics of the zebrafish *apoA-IV* genes, as measured by ISH, during somitogenesis [15–20 somite (S)] and at 1–6 dpf. All four *apoA-IV* genes showed earliest mRNA expression at 15–20 S in the YSL; the mRNAs localize to distinct subregions of the YSL until 5 dpf. All four genes are expressed in the intestine (I) at 4–6 dpf, with weak intestinal expression observed for *apoA-IVa*, and *apoA-IVb.3* is expressed in the liver (L) at 4–6 dpf. No expression was observed for the *apoA-IV* genes at the eight-cell stage, 30% epiboly or 80–100% epiboly (supplementary material Fig. S4). Larvae from 15–20 S to 5 dpf are wild type (2–5 dpf treated with PTU; 6-dpf larvae are nacre^−/−^. Experiments were performed in triplicate with *n*≥5 embryos or larvae per probe in every experiment.

*In-situ* hybridization (ISH) was carried out with antisense and control sense riboprobes specific to each apolipoprotein studied (Figs 2–5; supplementary material Figs S2–S6). All 11 apolipoproteins were expressed in the yolk syncytial layer (YSL) during embryogenesis (Figs 2–5), a finding consistent with the unique role of the YSL in processing yolk lipids and secreting VLDL to support embryonic and larval development (Carvalho and Heisenberg, 2010). The earliest apolipoprotein expression was observed for *apoEb* mRNA which localized to the YSL during blastulation (30% epiboly) (Fig. 4). Gastrulation (80–100% epiboly) is associated with the earliest expression of *apoA-Ia* and *apoA-Ib* mRNA in the YSL (Fig. 2). *apoBa*, *apoBb.1* (Fig. 3A), *apoEa* (Fig. 4) and the four *apoA-IV* genes (Fig. 5) are first expressed during somitogenesis (15–20 somites). Although many apolipoproteins are expressed uniformly throughout the YSL, some localize to specific YSL regions or in patterns indicative of subcellular niches. For example, at 80–100% epiboly, both *apoA-Ia* and *apoA-Ib* mRNA localized to perinuclear YSL regions (Fig. 2); at 15–20 somites, *apoBb.2* and *apoEa* were expressed strongly in distinct subregions of the YSL (Fig. 3A; Fig. 4); and at 1 dpf, *apoA-IVa*, *apoA-IVb.2* and *apoA-IVb.3* localize more strongly to the yolk extension than to the other areas of the YSL (Fig. 5).

As the digestive organs develop (4–6 dpf), the apolipoproteins examined begin to be expressed in the intestine and/or liver, often with gene paralogs segregated to different organs. ISH showed that *apoA-Ia* mRNA was expressed only in the intestine and that the majority of *apoA-Ib* mRNA localized to the liver, with only weak expression in the intestine (Fig. 2). The *apoB* genes showed similar segregated mRNA localization: *apoBa* was expressed in the heart (2 dpf), strongly in the liver (4–6 dpf) and weakly in the intestine (5–6 dpf), *apoBb.1* was expressed predominantly in the intestine and weakly in the liver (5–6 dpf), and *apoBb.2* was not expressed in the liver or intestine (Fig. 3A). mRNA of both *apoE* and all four *apoA-IV* genes localized to the intestine (*apoEa* at 6 dpf, all others 4–6 dpf) (Figs 4,5). The only *apoA-IV* paralog transcribed in both the liver and intestine is *apoA-IVb.1* (Fig. 5). *apoEa* and *apoEb* are expressed outside the YSL and digestive organs; *apoEb* localizes to the tail bud (15–20 somites through 1 dpf), apical ectodermal ridge (AER) (15–20 somites through 6 dpf), olfactory orifices (1–6 dpf), fin buds (2–6 dpf), esophagus and pharynx (2–6 dpf), gill arches (3–6 dpf), macrophages in the head (3–6 dpf) (Herbomel et al., 2001), retina (4–6 dpf), periderm (4–6 dpf), and both *apoE* genes are expressed in the swim bladder (6 dpf) (Fig. 4).

Through ISH we were able to qualitatively characterize spatiotemporal mRNA expression and localization of the zebrafish *apoB* paralogs. However, mRNA expression levels do not necessarily correlate with protein levels. Therefore, we used liquid chromatography tandem mass spectrometry (LC-MS/MS) to determine whether the *apoB* mRNA expression observed by ISH is mirrored by ApoB protein levels. Total larval proteins ≥250 kDa were analyzed (all three zebrafish ApoB paralogs run in this size range on a SDS-PAGE gel; I.A.D., H.T. and K.Y., unpublished observation). LC-MS/MS revealed that ApoBb.1 accounts for the majority of total ApoB protein in both 2- and 15-dpf larvae (96.3% of total ApoB protein at 2 dpf and 94.2% at 15 dpf) (Fig. 3B). ApoBa protein accounts for a small percentage of total ApoB at both developmental time points (3.1% of total ApoB protein at 2 dpf and 3.4% at 15 dpf) (Fig. 3B). Finally, at 2 dpf, ApoBb.2 represents extremely little of the total ApoB protein (0.6% of total ApoB protein), but by 15 dpf, it composes 2.4% of total larval ApoB protein (Fig. 3B).

### A high-fat feed induces apolipoprotein transcription

**Fig. 6.**
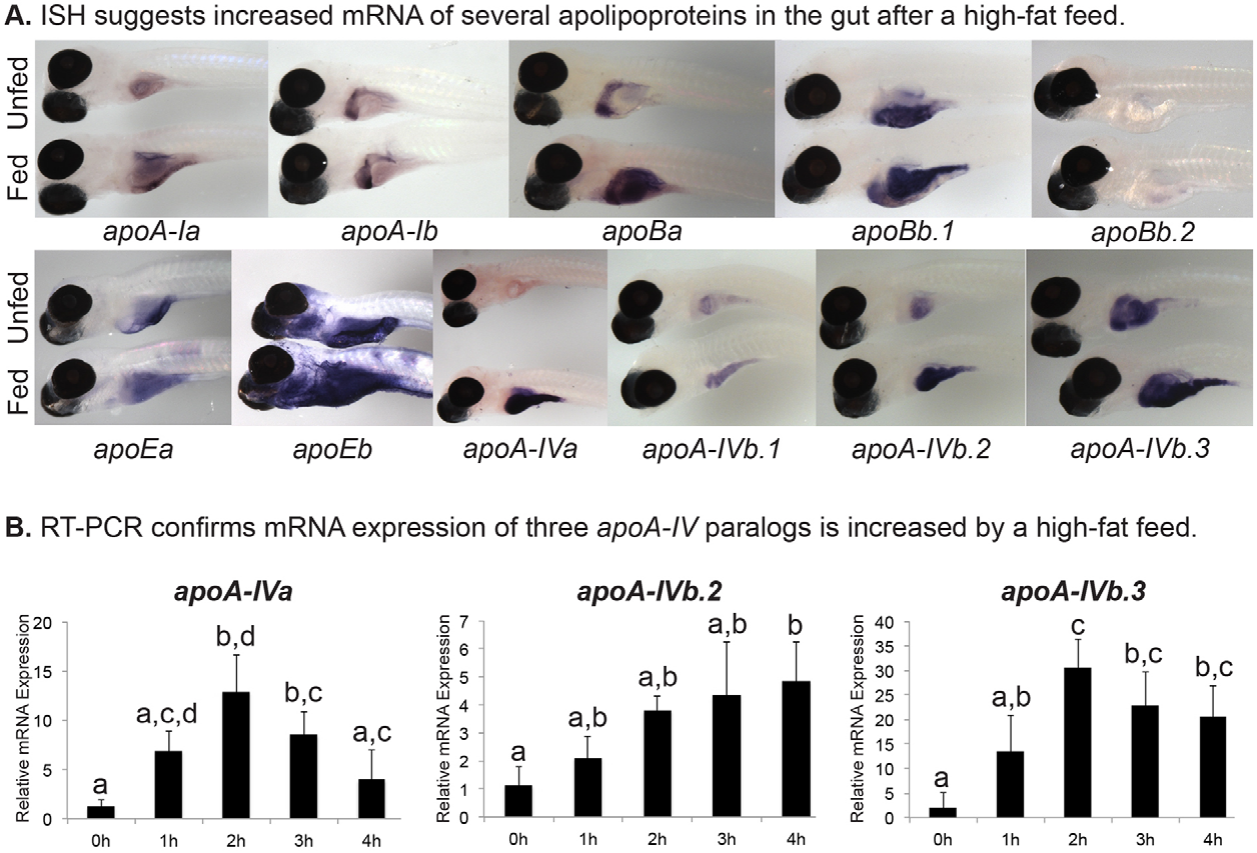
**High-fat feed increases expression of apolipoproteins in larval gut.** (A) Representative images of mRNA expression as observed by ISH of the zebrafish *apoA-I*, *apoB*, *apoE* and *apoA-IV* genes in 6-dpf nacre^−/−^ larvae. Larvae have either never eaten exogenous food (Unfed), or have been fed a high-fat, 5% chicken egg yolk meal for 4 hours (Fed). ISH was performed in triplicate with *n*≥5 larvae; no staining was observed in sense probed (supplementary material Figs S1–S4). (B) A high-fat meal increases zebrafish *apoA-IVa*, *apoA-IVb.2*, and *apoA-IVb.3* mRNA expression in the gut (intestine, liver, pancreas). Real-time PCR quantification of apolipoprotein transcriptional response in the 6-dpf zebrafish gut to 1, 2, 3 or 4 hours of a 10% chicken egg yolk feed (*n*=3; 10 pooled larval guts per experiment). Groups with the same letter are not significantly different (one-way ANOVA, *P*<0.05, *n*=3).

The presence in the liver and/or intestine of every apolipoprotein examined, except *apoBb.2*, at the 6-dpf developmental point that coincides with exhaustion of yolk nutrients and initiation of exogenous food intake, suggests that there is a crucial role for these proteins in dietary lipid transport. Despite this correlation, it is currently unknown whether ingestion of dietary lipids transcriptionally regulates zebrafish apolipoprotein mRNA expression. We predicted that dietary lipids might induce apolipoprotein expression given that the presence of these proteins is essential for lipid processing and transport. To address the hypothesis that ingestion of dietary lipids would induce apolipoprotein transcription, we used an established high-fat feeding paradigm in which chicken egg yolk emulsified in embryo medium (5% chicken egg yolk; 4 hours) is fed to larval zebrafish (Carten et al., 2011). Initially, we performed ISH on unfed and high-fat fed, 6-dpf larvae. Although ISH is a powerful tool to assess the localization of transcripts, it can only provide a qualitative measure of expression levels because the development of the colorimetric readout is a non-linear process. Despite this limitation, we observed increased intestinal and hepatic signals for some apolipoproteins following a high-fat feed, consistent with increases in mRNA expression (Fig. 6A).

To quantitatively assess the effect of dietary lipids on larval apolipoprotein mRNA expression, we performed quantitative real-time PCR (qRT-PCR) on the guts (intestine, liver and pancreas) of 6-dpf unfed larvae and their fed clutch-mates at 1, 2, 3 and 4 hours after the onset of a high-fat feed (10% chicken egg yolk; 1 hour) (Fig. 6B). qRT-PCR revealed a remarkable, ~30-fold increase in *apoA-IVb.3* mRNA at 2 hours following the onset of feeding (one-way ANOVA, *F*(4,10)=9.359, *P*=0.0021], a ~12-fold increase in *apoA-IVa* at the same time point [one-way ANOVA, *F*(4,10)=9.318, *P*=0.0021], and an ~5-fold increase in *apoA-IVb.2* by 4 hours post-feed onset [one-way ANOVA, *F*(4,10)=5.433, *P*=0.0137]. The increases in gene expression appear to be specific to ingestion of dietary lipids, as no changes were observed after a high-protein, low-lipid feed (10% chicken egg white; E.M.Z. and S.A.F., unpublished observation). In contrast, there were no significant changes in expression of the other apolipoproteins (supplementary material Fig. S7). RT-PCR was not performed for apoBb.2 as the expression level in the gut at 6 dpf is below the detection limit of these assays.

### Zebrafish ApoA-IV overexpression reduces high-fat food intake

Understanding the gut to brain signals underlying appetite regulation is crucial in the context of the current obesity epidemic. Rat APOA-IV is secreted from the intestine on chylomicrons following a high-fat feed and acts as a satiety factor, signaling via vagal afferents to centrally decrease food intake (Fujimoto et al., 1992; Fujimoto et al., 1993a). Therefore we hypothesized that ApoA-IV might have a conserved role in the regulation of food intake in zebrafish, predicting that it would decrease food intake during high-fat feeding. To address this question, we created transgenic zebrafish that overexpress ApoA-IVb.1 transiently under an inducible heat shock protein 70 (hsp70) promoter with a fluorescent reporter [*Tg(hsp70:apoA-IVb.1:mCherry)*].

**Fig. 7.**
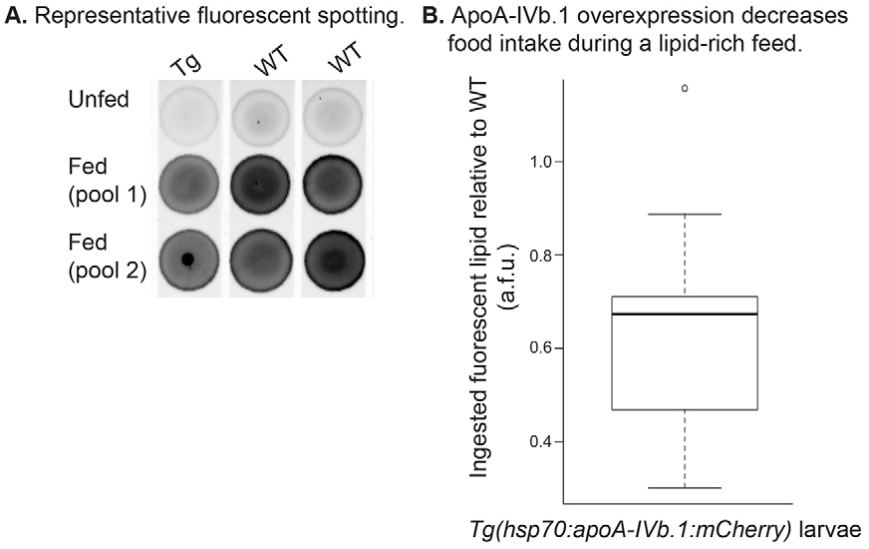
**Overexpression of zebrafish ApoA-IVb.1 decreases food intake.** (A) Representative fluorescent images of spotted lipid extracts; each spot represents the fluorescent lipids of 10 pooled larvae following lipid extraction. Tg represents *Tg(hsp70:apoA-IVb.1:mCherry)* larvae and WT represents wild-type larvae. Larvae have either never eaten exogenous food (Unfed), or have been fed a high-fat, 10% chicken egg yolk with 6.4 μM BODIPY® FL C_16_ for 4 hours (Fed). (B) *Tg(hsp70:apoA-IVb.1:mCherry)* larvae ingest fewer fluorescently labeled lipids as shown by comparison of arbitrary fluorescent units (a.f.u.) to wild type (paired Student’s *t*-test, *P*<0.001; *n*=9, 20 larvae per experiment). Total larval lipids were extracted and lipid fluorescence was measured. Natural fluorescent lipid background was corrected using measurements of unfed larvae. The box represents the 25–75th percentiles, and the median is indicated. The whiskers show the 10–90th percentiles.

We anticipated that if ApoA-IVb.1 induces satiety, *Tg(hsp70:apoA-IVb.1:mCherry)* larvae overexpressing ApoA-IVb.1 following a heat shock would eat less during a high-fat feed. To measure food intake, larvae were fed a high-fat meal (10% egg yolk; 4 hours) containing a fluorescently labeled lipid (BODIPY-C16), total larval lipids were extracted, and the amount of fluorescent lipids ingested was quantified as an indication of total food intake (Fig. 7A). Strikingly, larvae overexpressing ApoA-IVb.1 ingested approximately two-thirds of the fluorescent lipids compared with wild-type (WT) larvae (paired Student’s *t*-test, *P*=0.004) (Fig. 7B), demonstrating that ApoA-IV might impact food intake in zebrafish as it does in rats.

## DISCUSSION

### Gene ancestry analysis of zebrafish apolipoproteins

This is a comprehensive study of the zebrafish orthologs of four major human serum apolipoproteins that are associated with disease processes. Unifying the designations of zebrafish apolipoproteins will facilitate interpretation of results and translation to human physiology. Historically, naming zebrafish genes has been complicated by the extensive gene duplications that resulted from a whole genome duplication event in the teleost fish lineage as well as local gene duplication events (Amores et al., 1998; Gates et al., 1999; Postlethwait et al., 1998; Taylor et al., 2001). Furthermore, the zebrafish community only recently achieved a thorough annotation of the zebrafish genome (Howe et al., 2013). To ensure consistency between future studies and to facilitate translation to mammalian studies, we verified the ancestral relationships of the 11 zebrafish *apoA-I*, *apoB*, *apoE* and a*poA-IV* genes with their named human orthologs through sequence, syntenic and phylogenetic analyses.

This study and others have shown that zebrafish apolipoproteins tend to have low sequence similarity to their mammalian orthologs (supplementary material Fig. S1) (Babin et al., 1997; Durliat et al., 2000). Despite high sequence divergence, by integrating the results of our syntenic and phylogenetic analyses, we are confident that the zebrafish genes investigated in this study are accurately named after their human orthologs. The phylogenetic analysis is consistent with that of Babin et al., in that it is unable to confidently resolve the basal relationships between apolipoprotein families, but confidently groups zebrafish ApoA-I and ApoE into distinct clades with their vertebrate orthologs (Babin et al., 1997). The present phylogeny builds on previous findings by demonstrating that the zebrafish ApoB paralogs do share a phylogenetic clade with their human ortholog. The phylogenetic analysis also suggests that there are two subfamilies of zebrafish ApoA-IV genes that are not strongly linked by bootstrap support.

We expanded upon our phylogenetic analysis of the apolipoproteins by performing an analysis of syntenic gene regions in zebrafish and human orthologs. These results clearly show the effects of the teleost genome duplication, as there are two paralogs of zebrafish *apoA-I* and *apoE* that share syntenic gene relationships with human *APOA-I* and *APOE*. The same is true for zebrafish *apoBa* and *apoBb.1*, and human *APOB*, but the lack of syntenic gene similarity between *apoBb.2* and human *APOB* suggest that this gene resulted from an independent local duplication event.

The zebrafish *apoA-IV* paralogs do not share syntenic gene regions with human *APOA-IV*, but do unexpectedly share syntenic gene regions with human *APOE*. This led us to speculate that *APOE* and *APOA-IV* share a common ancestor. Indeed, coelacanth and several additional fish species contain a cluster of apolipoprotein genes that contain both *apoE* and a*poA-IV* (data not shown).

### Retention of duplicate apolipoproteins

It is noteworthy that zebrafish have retained multiple apolipoprotein paralogs over millions of years of evolution. The duplication-degeneration-complementation (DDC) model (Force et al., 1999) proposes an explanation of why duplicated genes might be retained. The DDC model predicts that, instead of being retained due to the evolution of novel functions (neofunctionalization), degenerative mutations in the regulatory elements of duplicated genes increase the likelihood of their preservation owing to conservation and partitioning of their ancestral functions (subfunctionalization). The zebrafish paralogs of *pax6* and the *igf1r* are clear examples of genes that have subfunctionalized. The paralogs of these genes perform similar functions in different tissues due to differential expression patterns, likely resulting, as the DDC model predicts, from mutations in regulatory elements (Kleinjan et al., 2008; Schlueter et al., 2006). We speculate that similar subfunctionalization has occurred in several of the zebrafish apolipoprotein paralogs. For example, although mammalian *APOA-I* is expressed both in the intestine and liver, zebrafish *apoA-Ia* is expressed in the intestine and the majority of *apoA-Ib* expression is observed the liver. Based on the DDC model, we would predict that there are mutations in the regulatory regions of these genes that restrict their expression to certain tissues while maintaining their ancestral function. Similar subfunctionalization might have occurred in the zebrafish *apoA-IV* paralogs because all four are expressed in the larval intestine, but only *apoA-IVb.3* is expressed in the liver. By comparison, human *APOA-IV* is expressed in the intestine, but rat *ApoA-IV* is expressed in the liver and intestine, hence, the ancestral gene might have had both hepatic and intestinal expression.

Zebrafish and humans might have used unique strategies to achieve tissue-specific *APOB* gene expression. In zebrafish, it is possible that the zebrafish *apoB* paralogs have subfunctionalized, with *apoBa* restricted to performing the function of the ancestral gene in the liver and heart [where it is expressed in humans and mice (Nielsen et al., 1998)] and *apoBb.1* performing the majority of function of the ancestral gene in the intestine. By contrast, tissue-specific human *APOB* expression occurs in the form of different isoforms of the same gene through mRNA editing; a short isoform, *APOB-48*, is expressed in the intestine, and a long isoform, *APOB-100*, is expressed in the liver. Although *apoBb.2* is the shortest zebrafish paralog, it is not a C-terminal truncation like human *APOB-48*, in that it contains numerous deletions throughout the protein (data not shown) and it is not expressed in the larval intestine. Zebrafish ApoB protein analysis by LC-MS/MS indicated higher protein levels of ApoBb.1 as compared to ApoBa or ApoBb.2. Therefore ApoBb.1 might have a greater role in lipid transport, but it is not clear whether higher expression of the ApoBb.1 protein provides a functional benefit in lipid transport as compared to the other paralogs. The mechanisms underlying the large differences in zebrafish ApoB protein levels have yet to be determined; however, unpublished RNAseq data of 6-dpf larval guts (E.M.Z. and S.A.F., unpublished observations) similarly shows much higher mRNA levels for *apoBb.1* than for *apoBa* or *apoBb.2*, suggesting that this regulation takes place at the mRNA level. It is difficult to postulate how the differences in protein levels between zebrafish ApoB paralogs compare to those of the human APOB isoforms as little data describing whole-body human APOB protein levels are available; however, Poapst el al. found that there is a similar serum protein mass of APOB-48 and APOB-100 in humans (Poapst et al., 1987).

The zebrafish *apoE* paralogs might also have undergone subfunctionalization. Here, we show *apoEa* localizing to the YSL and intestine, and both this and other studies have found widespread expression of *apoEb* (Babin et al., 1997; Herbomel et al., 2001; Monnot et al., 1999). Based on its spatiotemporal expression and localization patterns, we predict ApoEb likely retains the non-lipid transport, extra-digestive organ functions of the ancestral gene. Zebrafish *apoEb* has similar developmental expression to mouse *APOE* [visceral yolk sac endoderm at 8.5 days post-conception (dpc), liver, lung, heart and eye at 9.5 dpc, and cells lining the olfactory vesicle, peripheral neural cells, and the brain at 10.5 dpc (Bächner et al., 1999; Harrison et al., 1995)]. Given that both zebrafish *apoE* paralogs are expressed in the YSL, it is unclear from our data whether they exhibit different lipid transport properties. Monnot et al. (Monnot et al., 1999) observed similar expression of an unspecified *apoE* paralog during zebrafish development, as well as induction during adult fin regeneration.

The segregated expression of several larval apolipoprotein paralogs between organs provides a unique opportunity to study potential differences in lipoproteins generated from various tissues. Recent studies have emphasized the contribution of HDL particle composition, as opposed to solely HDL cholesterol levels, to lipoprotein function (Feig et al., 2014; Khera et al., 2011). It is not known whether nascent mammalian HDL particles synthesized by the liver have compositions that are unique to those assembled by the intestine. It is difficult to address this question with traditional mouse models because it is technically challenging to identify HDL particles of different origins. Promisingly, the nearly segregated expression patterns of the zebrafish *apoA-I* paralogs provide a system to study HDL particles of hepatic versus intestinal origins. The apolipoprotein mRNA localization data presented in this study does not show localization to any new organs compared to human ortholog expression, but functional studies will need to be performed in the future to rule out the possibility that any neofunctionalization has occurred.

### mRNA localization suggests important roles for apolipoproteins in YSL and dietary lipid transport

Previous ISH studies support our finding that nearly all zebrafish apolipoproteins show mRNA expression in the YSL (Babin et al., 1997; Thisse et al., 2001). The YSL is a syncytium containing many highly dynamic nuclei that form at the surface of the yolk cell during blastulation (Carvalho and Heisenberg, 2010). In addition to a multitude of key developmental functions, the YSL serves nutritive functions in the developing embryo, including the hydrolysis and export of yolk lipids in lipoproteins. Electron microscopy studies have visualized the secretion of VLDL particles from the highly developed secretary pathways of the fish YSL (Mani-Ponset et al., 1996; Walzer and Schönenberger, 1979) and studies in zebrafish lacking functional *mtp* (an *apoB* chaperone required for VLDL formation) have demonstrated the necessity of lipoprotein for export of yolk lipids (Miyares et al., 2014). Given these observations, it was expected that the zebrafish orthologs of mammalian apolipoproteins known to be associated with VLDL and chylomicrons (*apoB*, *apoE* and *apoA-IV*) would be expressed in the YSL. However, both *apoA-I* paralogs were also expressed in the YSL, similar to a previous report (Babin et al., 1997). The presence of *apoA-I* suggests that the YSL might produce HDL particles in addition to the classically described VLDL particles, or that the previously described VLDL particles have an apolipoprotein composition unique from classical VLDL, but similar to chylomicrons, which contain APOA-I. To our knowledge, characterization of the apolipoprotein composition of YSL lipoproteins has not been reported; these studies would have the potential to reveal a wealth of information regarding lipoprotein function during embryonic development.

Mammalian embryos also have a yolk sac in early development, prior to the development of a circulatory system. Like the fish YSL, the mammalian yolk sac secretes VLDL (Farese et al., 1995; Terasawa et al., 1999). It is likely that mammalian yolk sac apolipoproteins provide required nutritive contributions during embryogenesis, as *ApoB* (Terasawa et al., 1999) and *ApoE* (Harrison et al., 1995) mRNA are expressed in the mouse yolk sac and loss of *ApoB* (Farese et al., 1995) or *Mttp* (Raabe et al., 1998) is embryonic lethal. Knockdown of YSL lipoproteins is similarly lethal in zebrafish (Avraham-Davidi et al., 2012; Miyares et al., 2014). Taken together, these findings suggest that future studies of zebrafish YSL lipid metabolism can serve as an informative model of mammalian embryonic metabolism.

A second prominent finding from our spatiotemporal apolipoprotein mRNA expression analysis was that over half of the apolipoproteins were not distributed evenly throughout the YSL, but localized to distinct subregions. A cursory analysis of publically available YSL expression patterns on ZFIN reveals many additional examples of regional YSL gene expression, yet the significance of these YSL subregions, especially in relationship to embryonic lipid metabolism, has yet to be explored. Notably, the apolipoproteins do not adhere to the YSL expression patterns of *gata5* and *gata6*, markers of the presumptive YSL regions that will become the liver and intestine (Thisse et al., 2001). This supports the theory that apolipoprotein mRNA is translated into proteins that perform YSL-specific functions, and that the proteins are not simply being recruited to the nascent digestive tract.

### Dietary lipids induce apolipoprotein expression

Extensive research has elucidated much of the complex, molecular network that regulates transcription of the major serum apolipoproteins (Zannis et al., 2001), but it is less clear how the presence of dietary nutrients regulates apolipoprotein transcription. Dietary fats increase transcription of mammalian *ApoB* and *ApoA-IV*, two apolipoproteins involved in chylomicron assembly (Hernández Vallejo et al., 2009). *ApoA-IV* is very sensitive to nutritional status; dietary lipids increase intestinal *ApoA-IV* expression through HNF-4 promoter binding (Carrière et al., 2005). While fasting (Hanniman et al., 2006; LeBoeuf et al., 1994), high-fat diet (Hernández Vallejo et al., 2009), diabetes (Hanniman et al., 2006), and hepatic steatosis increase hepatic *ApoA-IV* expression through CREBH (Xu et al., 2014). Our results indicate that intestinal and hepatic mRNA expression of three of the four zebrafish *apoA-IV* paralogs is similarly increased in response to high-fat feeding, but whether the molecular mechanisms of this response mirrors those of mammals remains open to investigation.

In contrast to *ApoA-IV*, the effects of dietary lipids on intestinal and hepatic *ApoA-I* and *ApoE* transcription are less well studied. Additional questions regarding the effects of dietary lipids on apolipoprotein expression are raised by the paradoxical finding of Yoshioka et al. (Yoshioka et al., 2008) that a high-fat meal decreases mRNA expression of *ApoB* and one isoform of *ApoA-IV* in intestinal mucosa, while increasing mRNA expression of a second *ApoA-IV* isoform. It is possible that the inconsistent results found in various mouse models are due in part to the nutritional status of the animals. The mice in previous studies were fasted for various lengths of time prior to refeeding with high-fat diets of varying compositions and durations. An advantage of the larval zebrafish model is that the larvae used in this study were experiencing their first exogenous meal, previously subsisting on yolk nutrients. Therefore, the larval zebrafish offers a unique opportunity to study food intake in an animal that has homogeneity, which is often achieved by fasting animals, without the effects of starvation and refeeding. The zebrafish presents an exceptional opportunity to explore the potential synergistic effects of developmental stage on dietary lipid regulation of apolipoprotein transcription.

### Zebrafish apoA-IV decreases high-fat food intake

It has long been appreciated that APOA-IV is secreted from the intestine as a component of chylomicrons in response to long-chain dietary fatty acids (Hayashi et al., 1990; Kalogeris et al., 1996). Over 20 years ago, Fujimoto et al. (Fujimoto et al., 1992; Fujimoto et al., 1993b) demonstrated that acute, peripheral administration of APOA-IV decreases food intake in rats due to reduced meal size. Recent work supports the hypothesis that peripheral APOA-IV signals through the vagal nerve (Kalogeris et al., 1999; Lo et al., 2012) to the CNS [where *ApoA-IV* is expressed in the hypothalamus (Liu et al., 2001)] to decrease food intake (Fujimoto et al., 1993a; Shen et al., 2008). Recently, Wang et al. (Wang et al., 2013a) identified a specific portion of the N-terminal region of rat APOA-IV that is capable of decreasing food intake.

In this investigation, we demonstrated that overexpression of zebrafish ApoA-IV can decrease high-fat food intake. We developed a novel assay that measures total ingested larval fluorescent lipids (by lipid extraction, spotting on a TLC plate and quantification of total fluorescence) following a high-fat feed as an indicator of dietary lipid intake. This assay was used to demonstrate that acute overexpression of ApoA-IVb.1 decreases ingestion of fluorescently labeled C16 during a 4-hour high-fat feed in 7-dpf larvae. It is likely that some of the ingested BODIPY-C16 is metabolized into complex lipids, as we have previously demonstrated occurs with BODIPY-C16 fed to 6-dpf larvae (Carten et al., 2011) and BODIPY-C12 injected into the yolk of 3-dpf larvae (Miyares et al., 2014). However, metabolism of the BODIPY-C16 should not affect quantification of fluorescent lipid intake because total larval lipids were extracted and spotted on the TLC plate. Additionally, we do not believe that excretion of ingested lipids might have confounded our measurements of lipid uptake during the 4-hour feed. Prior studies of intestinal transit rates suggest that it would take >4 hours for excretion to commence: >3 hours is required for 7-dpf larvae to transport fluorescent microspheres to the distal intestine post-gavage and <10% of larvae transported the microspheres to the distal intestine by 6 hours post-gavage (Cocchiaro and Rawls, 2013).

It is possible that the additional zebrafish ApoA-IV paralogs also regulate food intake: they are highly transcriptionally responsive to dietary lipids, and ApoA-IVb.2 and ApoA-IVb.3 share a high degree of sequence similarity to ApoA-IVb.1 (supplementary material Fig. S1). Although ApoA-IVa has the most divergent sequence from ApoA-IVb.1, it is also highly induced by a high-fat feed. Hence, ApoA-IVa might share a role in regulating food intake, or, given that mammalian APOA-IV facilitates the production of smaller, more quickly cleared chylomicrons (Kohan et al., 2012), it might be produced to structurally support chylomicrons.

Unexpectedly, even though ApoA-IVb.1 overexpression decreased food intake, we were not able to detect mRNA expression in the CNS, where it is thought to regulate mammalian food intake. Therefore, it is possible that the signaling pathway by which ApoA-IVb.1 reduces food intake in larvae differs from that of adults. Alternatively, *apoA-IVb.1* might be expressed at an extremely low level in the CNS that ISH is not sensitive enough to detect. An alternative explanation of our results is that ApoA-IVb.1 signaling might vary by developmental stage. In fact, neonatal swine experience a high degree of *ApoA-IV* transcriptional induction by intraduodenal lipids before, but not after, weaning (Black et al., 1990). Future studies should define the degree to which endogenous zebrafish ApoA-IV proteins naturally regulate high-fat food intake and explore the signaling pathways by which this occurs.

Despite the relationship between acute APOA-IV administration and decreased food intake observed in rats, mice deficient in APOA-IV, or with chronically overexpressed APOA-IV, have no changes in food intake (Aalto-Setälä et al., 1994; Weinstock et al., 1997). The potentially conflicting results found in the mouse models of APOA-IV overexpression or loss could result from species differences between rats and mice. However, evidence showing that APOA-IV expression is decreased by a chronic high-fat diet and obesity (Tso and Liu, 2004), suggests that long-term overexpression or loss of APOA-IV might lead to loss of function. In our study, transient overexpression of zebrafish ApoA-IVb.1 by an inducible promoter decreased food intake during a lipid-rich feed. This finding supports a model where transient versus chronic induction of APOA-IV could have variable effects on food intake. Future studies could validate this model by creating transgenic zebrafish that chronically overexpress ApoA-IVb.1 on a constitutively active promoter, developing an assay to measure food intake in older larvae that have experienced long-term ApoA-IVb.1 overexpression, and determining whether regulation of lipid-rich food intake is lost.

### Conclusions

This study provides a framework for use in future studies of apolipoprotein biology, lipoprotein metabolism, dyslipidemias and the roles of apolipoproteins in development in the larval zebrafish. Presented here are analyses of the gene ancestry and genetic relationships between four major human serum apolipoproteins (APOA-I, APOB, APOE and APOA-IV) and their 11 zebrafish orthologs. The expression patterns of these zebrafish apolipoproteins were described throughout development and after a high-fat feed at 6 dpf. Finally, we determined that a zebrafish ortholog of ApoA-IV, an apolipoprotein that regulates high-fat food intake in rats, functions similarly in zebrafish. This comprehensive characterization of zebrafish apolipoproteins and the quantitative assay of larval lipid ingestion can serve as a foundation for future studies of the contributions of apolipoproteins to intestinal development and lipid processing in health and disease states, and in screens for pharmaceutical modulators of these proteins. Moreover, the quantitative feeding assay we developed can be applied to a wide range of studies investigating factors that regulate food intake. These data contribute to a growing literature establishing the larval zebrafish as a model for lipid metabolism in health and disease.

## MATERIALS AND METHODS

### Apolipoprotein gene ancestry analysis

To determine that zebrafish apolipoproteins share the same ancestral gene as their named human ortholog, we performed sequence, syntenic and phylogenetic analyses. The amino acid sequences of the zebrafish apolipoproteins were obtained from the Ensembl Genome Browser (Ensembl.org). First, the protein sequence of each zebrafish gene was used to query NCBI using BLASTp (standard settings) restricted to the human RefSeq protein database (Altschul et al., 1997; Altschul et al., 2005); the top BLAST hit for each query was recorded (Fig. 1A). Optimal pairwise alignments between zebrafish and human ortholog protein sequences were determined with the Needleman–Wunsch pairwise sequence alignment (Needleman and Wunsch, 1970) tool available at EMBL_EBI (http://www.ebi.ac.uk/Tools/psa/emboss_needle/) (McWilliam et al., 2013). Second, to examine the phylogenetic relationship between zebrafish apolipoprotein paralogs and their corresponding vertebrate orthologs, the amino acid sequences of human, zebrafish, mouse, chicken and coelacanth APOA-I, APOB, APOE and APOA-IV were aligned with using the iterative MAFFT multiple sequence alignment tool (Kuraku et al., 2013; Thompson et al., 2011). Specifically, the G-INS-I strategy was selected using standard settings, as it is recommended for sequences sharing global homology. Following alignment, the MAFFT tree server was used to build a neighbor-joining tree based on all gap-free sites, using the JTT substitution model and allowing estimation of site heterogeneity (alpha). Bootstrap resampling was set to 100, and bootstrap support ≥70 was interpreted as significant. Third, the syntenic relationships between the zebrafish and human apolipoproteins were established using the Genomicus (Louis et al., 2013) and SyntenyDB (Catchen et al., 2009) online syntenic analysis tools by searching for orthologous apolipoproteins in the corresponding syntenic region. Accession numbers of the zebrafish and human apolipoprotein genes used in this study are listed in supplementary material Table S1.

### Zebrafish

All procedures were approved by the Carnegie Institution Animal Care and Use Committee (protocol number 139) or the Weizmann Institute Animal Care and Use Committee (protocol number 01190212-3). For ISH experiments, WT (AB background) and melanophore-free (nacre^−/−^) (Lister et al., 1999) embryos were collected from natural spawning and were staged and raised in zebrafish embryo medium (EM) as described previously (Kimmel et al., 1995; Westerfield, 1995). WT larvae older than 1 dpf used for ISH experiments were treated with 0.003% N-phenylthiourea (PTU) (Sigma, St Louis, MO, USA) (Karlsson et al., 2001) or hydrogen peroxide (Thisse and Thisse, 2008) to decrease pigmentation. Zebrafish were fixed in 4% paraformaldehyde (PFA) in phosphate-buffered saline overnight at 4°C, washed twice in methanol, and stored in methanol at −20°C. Larvae raised to 15 dpf for ApoB protein measurements were fed three times per day from 7–15 dpf [larval AP100 food is composed of marine, animal and vegetable proteins (50% minimum protein content), yeast, vegetable starches, fish and vegetable oils, vitamin and mineral premixes, pigments, antioxidants and biodegradable binders (larval AP100, number 384709-20, Zeigler, Gardners, PA)].

### Preparation of feeding liposomes

Our laboratory and others have developed a high-fat feeding paradigm for larvae using chicken egg yolk (Carten et al., 2011; Marza et al., 2005). Briefly, 5% and 10% chicken egg yolk emulsions were prepared into liposomes from frozen aliquots of egg yolk resuspended in EM as previously described (Carten et al., 2011). The egg yolk emulsion was vortexed (2 minutes) and pulse sonicated with a one-quarter inch tapered microtip (5 seconds total processing time, 1 second on, 1 second off; output intensity of 3W) for 40 seconds in a sonicator (Ultrasonic Processor 3000, Misonix Inc., Farmingdale, NY).

### Feeding assay for ISH

Larvae (nacre^−/−^, 6 dpf) were fed a high-fat meal of 5% egg yolk liposomes for 4 hours at 29°C on an incubated shaker (Incu-Shaker Mini, Benchmark, Edison, NJ). Fed larvae were washed three times in EM, anesthetized with tricaine (Argent Chemical Laboratories, Redmond, WA), screened for the presence of food in the intestine, and fixed in 4% PFA as described above. Unfed clutch-mates were treated in tandem in EM and collected as controls.

### Whole-mount ISH

To elucidate the spatiotemporal mRNA expression patterns of the zebrafish apolipoproteins, ISH was carried out as previously described (Thisse and Thisse, 2008). Briefly, ~400–800 base pairs of unique sequence (consisting of coding sequence and in some cases 5′ or 3′ untranslated regions) of *apoA-Ia*, *apoA-Ib*, *apoBa*, *apoBb.1*, *apoBb.2*, *apoEa*, *apoEb*, *apoA-IVa*, *apoA-IVb.1*, *apoA-IVb.2* and *apoA-IVb.3* were amplified from cDNA (Primers listed in supplementary material Table S2), TOPO^®^ cloned into pCRII (Invitrogen, Grand Island, NY), and used to generate sense and antisense digoxigenin-labeled riboprobes (digoxigenin was from Roche, Indianapolis, IN). The riboprobes were hybridized against zebrafish of the following stages: eight-cell (to examine the presence of maternal mRNA transcripts), 30% epiboly (blastula), 80–100% epiboly (gastrula), 15–20 somite (somitogenesis), 1 dpf, 2 dpf, 3 dpf, 4 dpf, 5 dpf, and unfed and fed 6 dpf. ISH experiments were carried out three times on *n*≥5 larvae, for each sense and antisense probe, at each stage.

### Feeding assay for qRT-PCR experiments

For assays measuring the transcriptional responses of zebrafish apolipoproteins to a lipid-rich feed, larvae (AB, 6 dpf, staged) were placed in a 10% egg yolk emulsion on an incubated shaker (29°C). After 1 hour of feeding, larvae were washed three times in EM, anesthetized with tricaine, and examined for full intestines. Larvae representing a 1-hour time point were set aside for dissection; remaining larvae were incubated at 29°C in EM for the remainder of the time course and collected for dissection at 2, 3 and 4 hours. Unfed clutch-mates were incubated in EM, anesthetized, and collected in parallel as controls. Larval guts (intestine, liver and pancreas) were dissected, pooled in groups of 10, transferred to 30 μl RNALater (Ambion, Grand Island, NY), and stored at −20°C. Pooled experimental groups were collected in triplicate from unique clutches.

### qRT-PCR sample preparation and analysis

RNA was extracted from larval guts using an RNAqueous Micro Kit (Ambion, Grand Island, NY) and stored at −80°C. cDNA was constructed using the iScript cDNA Synthesis Kit (Bio-Rad, Hercules, CA). qRT-PCR samples were prepared using cDNA, SsoAdvanced Univeral SYBR Green Supermix (Bio-Rad), and primers designed against gene-specific sequence (supplementary material Table S3). qRT-PCR was performed in triplicate for each sample on the Bio-Rad CFX96 Real-Time System with 45 cycles: 95°C for 15 seconds, 59°C for 20 seconds and 72°C for 20 seconds. Results were analyzed using the Bio-Rad CFX Manager 3.0 software platform. Following qRT-PCR, representative samples were run on a 1% agarose gel and stained with ethidium bromide to verify the absence of multiple amplicons (supplementary material Fig. S8). qRT-PCR gene expression results were quantified using the ΔΔCT method (Livak and Schmittgen, 2001); *rps18* was used as the reference gene.

### Total larval protein purification and in-gel digestion

Relative protein expression levels of the zebrafish apoB paralogs was measured by purifying total larval protein and performing LC-MS/MS. To purify total protein, 20–30 embryos (2 and 15 dpf) were anesthetized on ice, washed twice with buffer A (β-glycerophosphate 500 mM, EGTA 15 mM, orthovanadate 1 mM, EDTA 10 mM, benzamidine 1 mM, aprotinin 10 mg/ml, leupeptin 10 mg/ml, pepstatin 2 mg/ml) and suspended in 400 μl buffer H (buffer A supplemented with 1 mM DTT). Embryos were homogenized with a 27-gauge syringe and sonicated twice on ice for 7 seconds at 40% power. Samples were centrifuged at 20,800 *g* at 4°C for 15 minutes, and supernatants were collected.

Total larval proteins were separated by SDS-PAGE using a 6% separating gel [4.5 ml H_2_O, 1.7 ml 30% acrylamide, 2.1 ml separating buffer (3M Tris-HCl pH 8.8, APS 1:100, TEMED 1:1000)] and a 4% stacking gel [3 ml H_2_O, 0.66 ml 30% acrylamide, 1.25 ml separating buffer (0.5M Tris-HCl pH 6.9, APS 1:100, TEMED 1:1000)]. GelCode Blue Stain Reagent (24590, Thermo Scientific) was used for protein detections according to manufacturer’s instructions.

Selected areas of interest (250–550 kDa, supplementary material Fig. S6) were excised from the gel, sliced into small (1–2 mm) pieces and placed in a silicon tube (0.65 ml; PGC Scientific, Frederick, MD). Gel bands were destained with 25 mM NH_4_HCO_3_ in 50% acetonitrile and dried by centrifugation under vacuum. Protein disulfide bonds were reduced by saturating the dry gel bands with 10 mM DTT in 25 mM NH_4_HCO_3_ at 56°C for 1 hour, and were alkylated with 55 mM iodoacetamide in 25 mM NH_4_HCO_3_ in the dark for 45 minutes at room temperature. After washing in 25 mM NH_4_HCO_3_ in 50% acetonitrile and centrifugation under vacuum, proteins were digested by rehydrating the gel pieces with 12.5 ng/μl trypsin in 25 mM NH_4_HCO_3_ at 4°C for 10 minutes followed by overnight incubation at 37°C. Peptides were extracted by addition of 50% acetonitrile and 5% formic acid, vortexing, sonicating, centrifuging and collection of the supernatant. Digestions were stopped by addition of trifluroacetic acid (1%). Extracted proteins were stored at −80°C until further analysis.

### Liquid chromatography

ULC and MS grade solvents were used for all chromatographic steps. Sample were loaded using split-less nano-Ultra Performance Liquid Chromatography (10 kpsi nanoAcquity; Waters Corporation, Milford, MA). The mobile phase consisted of (A) H_2_O and 0.1% formic acid and (B) acetonitrile and 0.1% formic acid. Desalting of the samples was performed online using a reversed-phase C18 trapping column (180 μm internal diameter, 20 mm length, 5 μm particle size; Waters Corporation). Next, the peptides were separated using a T3 HSS nano-column (75 μm internal diameter, 250 mm length, 1.8 μm particle size; Waters Corporation) at 0.35 μl/minute. Peptides were eluted from the column into the mass spectrometer with the following gradient: 4% to 35% B in 105 minutes, 35% to 90% B in 5 minutes, maintained at 95% for 5 minutes and return to initial conditions.

### Mass spectrometry

The nanoUPLC was coupled online through a nanoESI emitter (10 μm tip; New Objective; Woburn, MA) to a quadruple orbitrap mass spectrometer (Q Exactive Plus, Thermo Scientific) using a FlexIon nanospray apparatus (Proxeon). Data was acquired in DDA mode, using a Top12 method. MS1 resolution was set to 60,000 (at 400 *m*/*z*) and maximum injection time was set to 120 milliseconds. MS2 resolution was set to 17,500 and maximum injection time of 60 ms.

### Data analysis of mass spectrometry experiments

Raw data was processed using Proteome Discoverer v1.41. The MS/MS spectra were searched using Mascot 2.5 (Matrix Sciences, London, UK) and Sequest HT against the zebrafish protein database UniprotKB (http://www.uniprot.org/) appended with 125 common laboratory contaminant proteins. A fixed modification was set to carbamidomethylation of cysteine and a variable modification was set to oxidation of methionine. Search results were then imported back to Expressions to annotate identified peaks. Proteins were grouped based on shared peptides and identifications were filtered such that the global false discovery rate was a maximum of 1%.

### Generation of transgenic zebrafish

Gateway cloning was used to create a construct expressing zebrafish ApoA-IVb.1 and mCherry under the heat shock protein 70 (hsp70) promoter and stable transgenic zebrafish lines were generated as previously reported (Kwan et al., 2007). Zebrafish *apoA-IVb.1* was amplified from cDNA (forward, 5′-GGGGACAAGTTTGTACAAAAAAGCAGGCTCCATGAAACTGTATCTGATA-3′; reverse, 5′-GGGGACCACTTTGTACAAGAAAGCTGGGTCTTAATATCTCTTGGTGA-3′) and cloned into a Gateway middle entry vector. We then used gateway cloning to create a *hsp70:apoA4b.1:mCherry* construct flanked by tol2 sites, which was injected into the yolk of 1–2-cell embryos with tol2 transposase for genome incorporation. Transgenic *Tg(hsp70:apoA-IVb.1:mCherry)* larvae were identified at 2 dpf by heat shocking larvae in 15 ml EM at 37°C for 45 minutes followed by 42°C for 10 minutes and screening for diffuse fluorescence throughout the entire larval body 18 hours later. Larvae were heat shocked a second time at 6 dpf, 18 hours prior to feeding assays, to induce ApoA4b.1 and mCherry overexpression. At 7 dpf, dim mCherry fluorescence was visible throughout the larvae at 10× magnification, and mCherry accumulation was visible in the vasculature, notochord and pronephros with confocal microscopy (supplementary material Fig. S9).

### Experimental feeding assays for *Tg(hsp70:apoA-IVb.1:mCherry)* larvae

As an indicator of food intake, *Tg(hsp70:apoA-IVb.1:mCherry)* and control WT larvae were fed liposomes labeled with BODIPY^®^ FL C_16_ (D-3821, Thermo Fisher Scientific, Inc., Rockville, MD), a green fluorescent fatty acid. Labeled liposomes were made by adding BODIPY^®^ FL C_16_ to 5 ml of 10% egg yolk emulsion immediately following sonication, for a final concentration of 6.4 μM BODIPY^®^ FL C_16_, and vortexing for 30 seconds. *Tg(hsp70:apoA-IVb.1:mCherry)* and WT larvae (7 dpf) were placed in the fluorescent liposome solution in an incubated shaker for 4 hours at 29°C. Larvae were washed three times in EM, anesthetized in tricaine and screened for the presence of food in the intestine. Groups of 10 larvae were pooled, flash frozen and stored at −80°C. Feeding assays were performed pairwise with one pool of 10 transgenic larvae and two pools of 10 WT larvae. Unfed, WT and transgenic larvae were treated in parallel and collected as controls. A total of nine paired sets of larvae were collected. All experimental and control Tg and WT larvae were treated with the same heat shock protocol at 2 and 6 dpf as described above.

### Lipid extraction and analysis

Total lipids were extracted from larvae that were fed fluorescent liposomes similarly to our previous report (Miyares et al., 2014) following the Bligh and Dyer method (Bligh and Dyer, 1959). Briefly, 100 μl of H_2_O was added to the frozen pool of 10 larvae and it was sonicated for 4 seconds with a one-quarter-inch inch tapered microtip with an output of 3W. Next, 375 μl of chloroform:methanol (1:2) was added to the homogenate, vortexed for 30 seconds and incubated at 25°C for at least 10 minutes. 125 μl of chloroform and 125 μl of 200 mM Tris-HCl pH 7, were subsequently added, with 30 seconds of vortexing after each addition. The samples were centrifuged at 1800 *g* for 5 minutes. The organic phase was transferred to a clean microfuge tube and stored at −80°C. For analysis, samples were dried in a speed vacuum, resuspended in 12 μl chloroform, and spotted on a channeled TLC plate (Whatman Scientific, Florham Park, NJ). To detect BODIPY^®^ FL C_16_ (as an indication of food intake), plates were scanned (Typhoon Scanner, GE Healthcare, Pittsburgh, PA) using a blue fluorescence laser (excitation: 488 nm; emission: 520 nm; band pass, PMT 425). Total fluorescence of each sample was quantified using ImageQuant software (GE Healthcare, Pittsburgh, PA, USA). Correction for naturally fluorescent lipid background was made with paired, unfed larval samples.

### Statistics

Differences in gene expression over the course of a 4-hour feed as measured by qRT-PCR were determined by one-way ANOVA followed by a Holm–Sidak test for multiple comparisons. Assays to compare difference in feeding between WT and *Tg(hsp70:apoA-IVb.1:mCherry)* larvae were performed pairwise with one sample of transgenic larvae and two samples of WT larvae per experiment. For each experiment, the fluorescence of the transgenic sample, expressed in arbitrary units, was normalized to the two WT samples averaged together. Difference in the amount of fluorescent lipids ingested between WT and *Tg(hsp70:apoA-IVb.1:mCherry)* larvae was analyzed by a paired Student’s *t*-test. Residuals of all the data were normally distributed before analysis and significance was set at *P*<0.05.

## Supplementary Material

Supplementary Material
