# Supplementary material for: Zebrafish as a model for apolipoprotein biology: comprehensive expression analysis and a role for ApoA-IV in regulating food intake
Source: Dis Model Mech. 2015 Jan 29;8(3):295–309. doi: 10.1242/dmm.018754 (PMC4348566; doi:10.1242/dmm.018754)
Supplement: Supplementary Material [file supp_8_3_295__index.html]

Zebrafish as a model for apolipoprotein biology: comprehensive expression analysis and a role for ApoA-IV in regulating food intake — Supplementary Material 

# Zebrafish as a model for apolipoprotein biology: comprehensive expression analysis and a role for ApoA-IV in regulating food intake

## DMM018754 Supplementary Material

**Files in this Data Supplement:**

- **Supplementary Material**
