# Supplementary material for: Zebrafish as a model for apolipoprotein biology: comprehensive expression analysis and a role for ApoA-IV in regulating food intake
Source: Dis Model Mech. 2015 Jan 29;8(3):295–309. doi: 10.1242/dmm.018754 (PMC4348566; doi:10.1242/dmm.018754)
Supplement: Supplementary Material [file supp_8.3.295_DMM018754.pdf]

Supplementary Materials

Figure S1. Sequence identity and similarity scores for zebrafish and human apolipoproteins. Percent similarity and identity between zebrafish and human orthologs was determined by ClustalW (v1.83) multiple sequence alignment with standard settings.

Figure S2. In situ hybridization analysis of *apoA-Ia* and *apoA-Ib* does not reveal maternal transcripts at the 8-cell (C) stage, and no induction of transcription at 30% epiboly (E). No staining is present in zebrafish treated with sense probes at any stage. Larvae are wild type (2–5 dpf treated with PTU) and nacre-/- (6 dpf). Experiments were performed in triplicate; n ≥ 5 larvae for each probe in every experiment. S: somite, dpf: days post-fertilization.

Figure S3. *apoBa*, *apoBb.1*, and *apoBb.2* in situ hybridization does not reveal mRNA transcripts at the 8-cell (C) stage, 30% epiboly (E), 80% E. No staining was observed for sense probes. Experiments were performed in triplicate on wild type (2–5 dpf treated with PTU) and nacre-/- (6 days post-fertilization (dpf)) larvae; n ≥ 5 larvae for each experiment.

Figure S4. *apoEa* has no or low expression in 8-cell (C), 30% epiboly (E), or 80% E larvae; a*poEb* mRNA is not present in 8-C embryos. Sense probes show no staining at any stage (S: somite). In situ hybridization (ISH) was performed in wild type and nacre-/- zebrafish. Larvae from 2 to 6 days post-fertilization (dpf) were treated with hydrogen peroxide. ISH was performed in triplicate; n ≥ 5 larvae for each experiment.

Figure S5. Zebrafish *apoA-IVa*, *apoA-IVb.1*, *apoA-IVb.2*, and *apoA-IVb.3* mRNA are not revealed at the 8-cell (C) stage, 30% epiboly (E), or 80% E, as measured by in situ hybridization (ISH). Experiments were performed in triplicate (n ≥ 5 larvae each) on wild type (2–5 dpf PTU treated) and nacre-/- (6 dpf). No staining was observed with sense probes for any of the *apoA-IV* genes at any stage. S: somite. Data for *apoA-IVb.1*, *apoA-IVb.2* sense and antisense probes and *apoA-IVb.3* sense probe at 30% E not shown.

Figure S6. SDS-PAGE gel of total larval protein from 2 and 15 days post-fertilization (dpf) larvae (20–30 pool larvae per sample) stained with GelCode. Red boxes represent the regions of the gel (≥ 250 kDa) excised for liquid chromatography mass spectrometry ApoB protein analysis.

Figure S7.

A high-fat meal does not change zebrafish *apoA-Ia*, *apoA-Ib*, *apoBa*, *apoB.1*, *apoEa*, *apoEb*, or *apoA-IVb.3* mRNA expression in the gut (intestine, liver, pancreas). Real-time PCR quantification of apolipoprotein transcriptional response in the 6-dpf zebrafish gut to 1, 2, 3, or 4 hours of a 10% chicken egg yolk feed (n = 3; 10 pooled larvae per n).

Figure S8.

Representative images of real-time PCR reactions run on 1% agarose gels to verify the presence of a single amplicon; n= 4 shown per primer set.

Figure S9.

Representative images of *Tg(hsp70:apoA-IVb.1:mCherry*) larvae ~18 hours post-heat shock. mCherry signal represents mCherry protein; mCherry signal in the digestive organ overview is visible in the pronephros. Larvae are 7 days post-fertilization, anesthetized in tricane and immobilized in 1.2% low melt agarose. Imaging was conducted with a 25X water immersion objective on a Leica SP5 confocal microscope. Scale bars represent 10 microns.

Figure S1.

Top Right: % Identity

	apoA-Ia (Zebrafish)	apoA-Ib (Zebrafish)	APOA-I (Human)	apoBa (Zebrafish)	apoBb.1 (Zebrafish)	apoBb.2 (Zebrafish)	APOB (Human)	apoEa (Zebrafish)	apoEb (Zebrafish)	APOE (Human)	apoA-IVa (Zebrafish)	apoA-IVb.1 (Zebrafish)	apoA-IVb.2 (Zebrafish)	apoA-IVb.3 (Zebrafish)	APOA-IV (Human)
apoA-Ia (Zebrafish)	100	38.5	49.1	2.4	2.7	2.9	2.2	19.6	18.5	18.9	45.5	52.7	52.3	52.3	34.5
apoA-Ib (Zebrafish)	61.5	100	45.5	2.2	2.4	2.5	1.9	17	15.6	15.7	37.7	46.2	45.5	46.2	31
APOA-I (Human)	24.7	22	100	2.4	2.6	3.3	2.2	18.7	20.9	18.6	42.7	47.2	46.4	46.8	32
apoBa (Zebrafish)	0.8	0.7	0.9	100	30.2	19.5	51.6	1	1	1	0.9	0.8	0.7	0.8	1
apoBb.1 (Zebrafish)	1.1	0.9	1	49.5	100	30.2	42.5	1	1	1.2	0.9	1.1	1	1	1.4
apoBb.2 (Zebrafish)	1.2	0.9	1.4	31.5	42.4	100	27.6	1	1.2	1	1.3	1.4	1.4	1.3	1.1
APOB (Human)	0.9	0.8	0.9	32	24.4	16.4	100	0.9	1.2	0.9	0.8	1.1	1.1	1	1.3
apoEa (Zebrafish)	45.5	40.4	41.4	2.6	2.8	3.2	2.4	100	43.1	23	41.5	49.5	50.2	50.9	32.4
apoEb (Zebrafish)	43.4	40.4	41.8	2.6	2.8	3.2	2.4	70.1	100	24.4	40.9	48.4	50.2	50.2	35.6
APOE (Human)	38.2	35.2	37.1	2.7	2.9	3.4	2.3	48.9	49.7	100	32.8	36.6	37.2	37.9	30.5
apoA-IVa (Zebrafish)	17.8	15.5	17.6	2.2	2.4	3.2	2.2	20.7	19.2	13.6	100	39.7	38.9	38.1	15.8
apoA-IVb.1 (Zebrafish)	24.6	20.3	26.2	2.4	2.8	3.4	2.3	22.7	22.4	18.6	66.5	100	93.3	91.4	19.1
apoA-IVb.2 (Zebrafish)	24.6	19.9	26.2	2.4	2.8	3.4	2.3	24.2	23.1	18.6	66.9	96.5	100	95.7	18.6
apoA-IVb.3 (Zebrafish)	24.2	20.7	24.3	2.5	2.8	3.4	2.3	24.2	21.7	18.6	66.5	96.1	98.8	100	18.3
APOA-IV (Human)	16.1	12.9	16.5	3.2	3.8	3.7	3.6	13.4	13.3	14.4	32.2	36.1	35.6	36.1	100

Bottom Left: % Similarity

Figure S2.

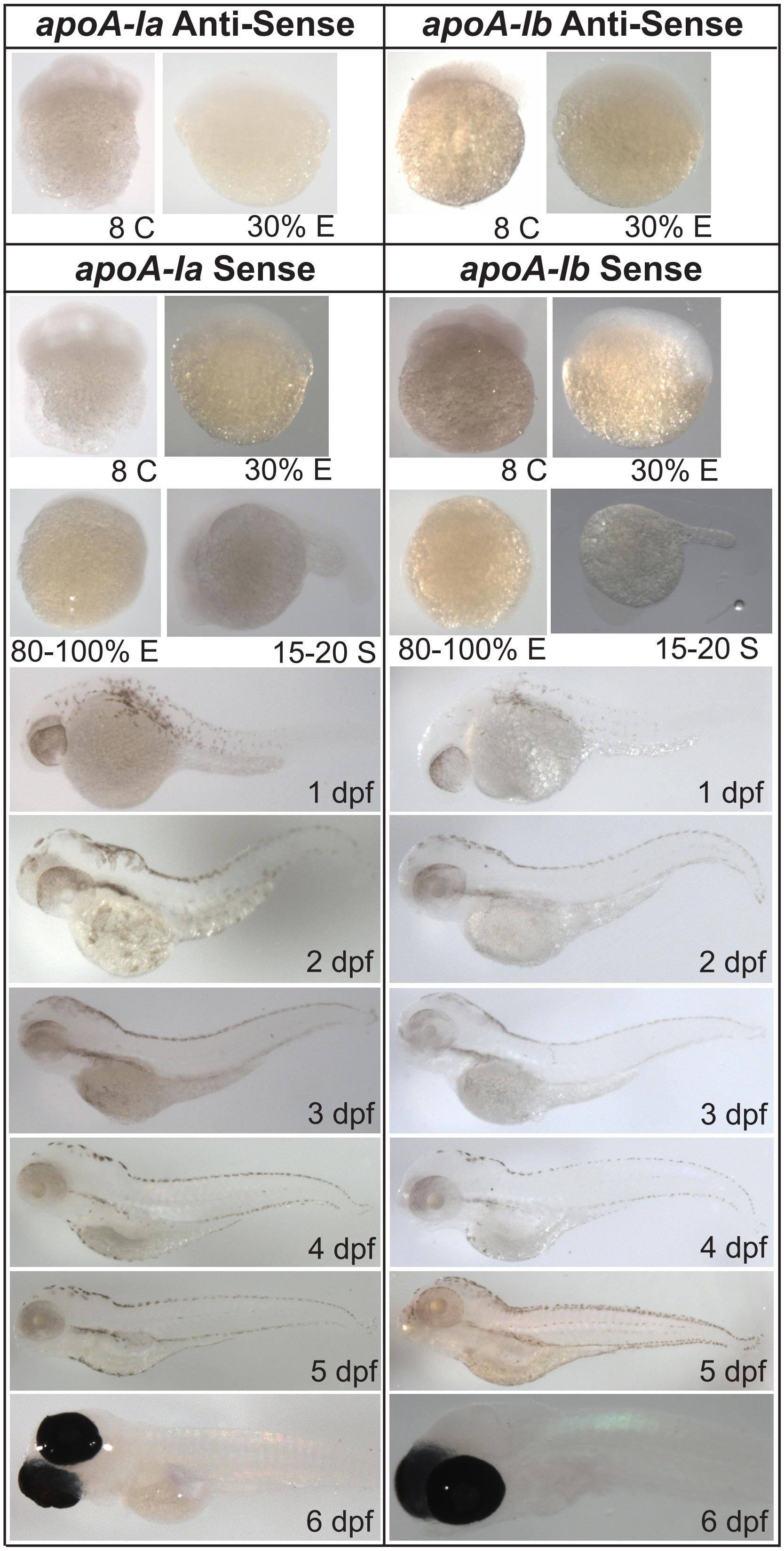

apoA-Ia Anti-Sense
apoA-Ib Anti-Sense
8 C
30% E
8 C
30% E
apoA-Ia Sense
apoA-Ib Sense
8 C
30% E
8 C
30% E
80-100% E
15-20 S
80-100% E
15-20 S
1 dpf
1 dpf
2 dpf
2 dpf
3 dpf
3 dpf
4 dpf
4 dpf
5 dpf
5 dpf
6 dpf
6 dpf

Figure S3.

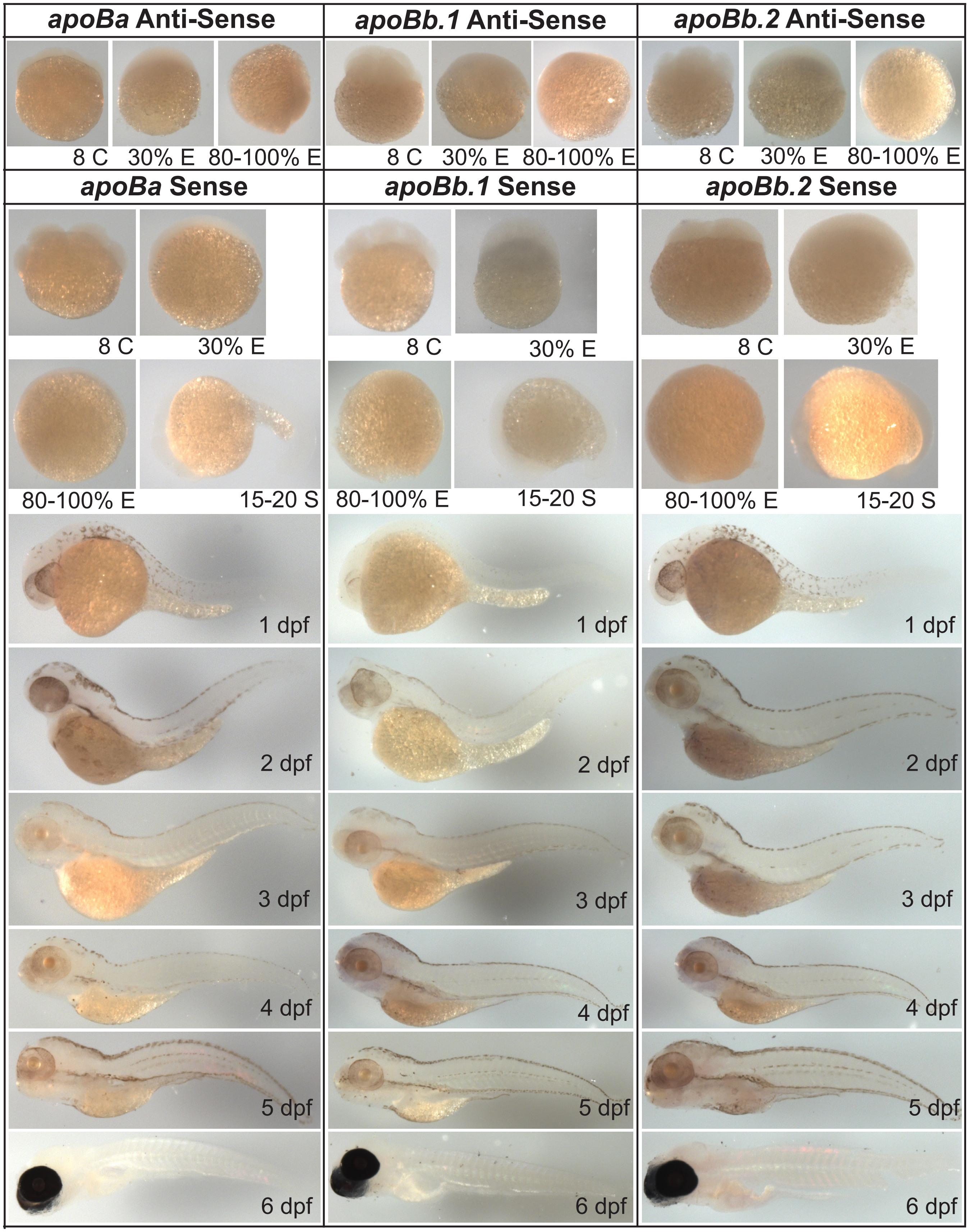

apoBa Anti-Sense
apoBb.1 Anti-Sense
apoBb.2 Anti-Sense
8 C
30% E
80-100% E
8 C
30% E
80-100% E
8 C
30% E
80-100% E
apoBa Sense
apoBb.1 Sense
apoBb.2 Sense
8 C
30% E
80-100% E
15-20 S
8 C
30% E
80-100% E
15-20 S
8 C
30% E
80-100% E
15-20 S
1 dpf
2 dpf
3 dpf
4 dpf
5 dpf
6 dpf
1 dpf
2 dpf
3 dpf
4 dpf
5 dpf
6 dpf
1 dpf
2 dpf
3 dpf
4 dpf
5 dpf
6 dpf

Figure S4.

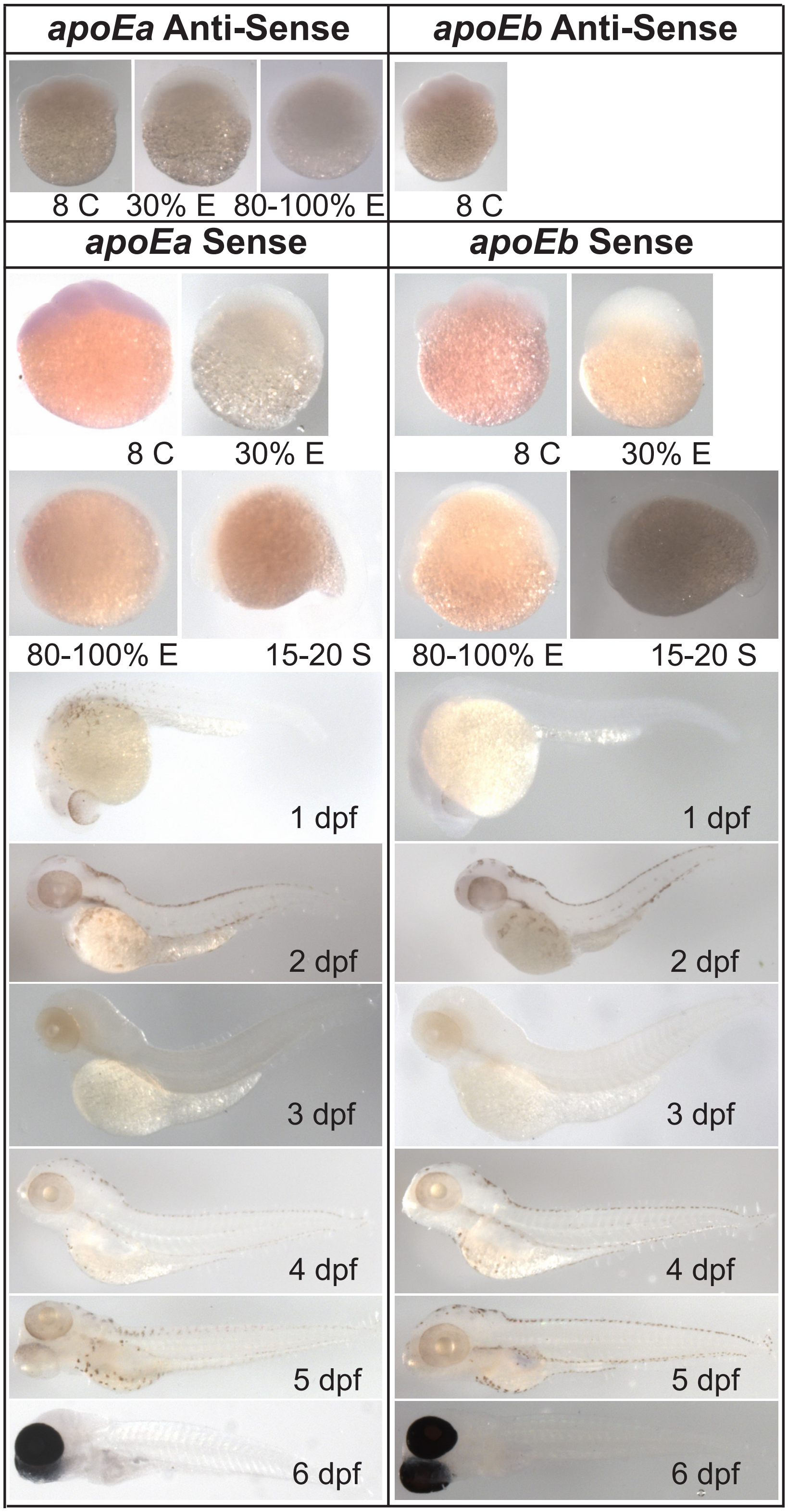
apoEa Anti-Sense
apoEb Anti-Sense
8 C
30% E
80-100% E
8 C
apoEa Sense
apoEb Sense
8 C
30% E
8 C
30% E
80-100% E
15-20 S
80-100% E
15-20 S
1 dpf
1 dpf
2 dpf
2 dpf
3 dpf
3 dpf
4 dpf
4 dpf
5 dpf
5 dpf
6 dpf
6 dpf

Figure S5.

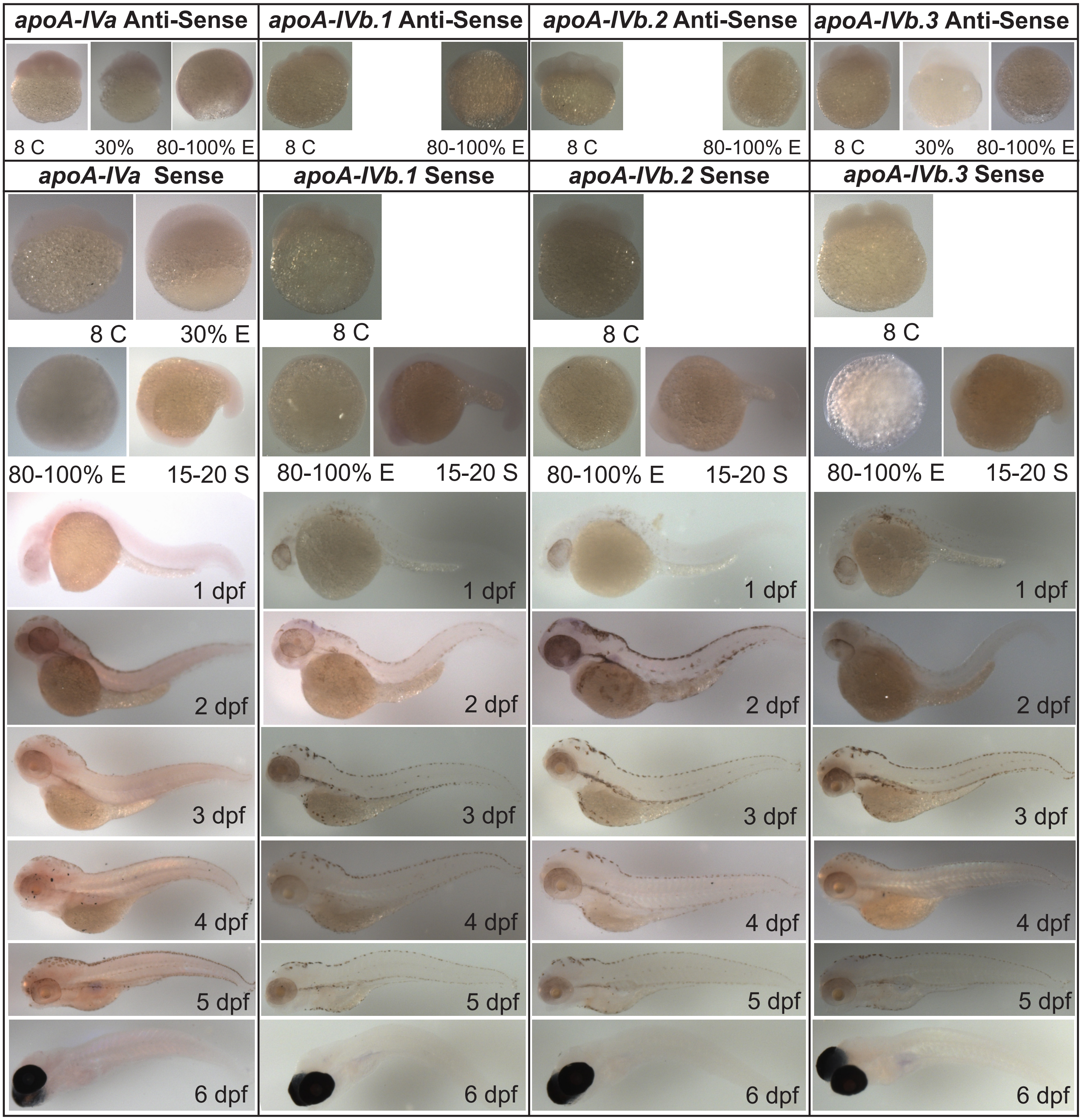
apoA-IVa Anti-Sense
apoA-IVb.1 Anti-Sense
apoA-IVb.2 Anti-Sense
apoA-IVb.3 Anti-Sense
8 C
30%
80-100% E
8 C
80-100% E
8 C
80-100% E
8 C
30%
80-100% E
apoA-IVa Sense
apoA-IVb.1 Sense
apoA-IVb.2 Sense
apoA-IVb.3 Sense
8 C
30% E
8 C
8 C
8 C
80-100% E
15-20 S
80-100% E
15-20 S
80-100% E
15-20 S
80-100% E
15-20 S
1 dpf
2 dpf
3 dpf
4 dpf
5 dpf
6 dpf

Figure S6.

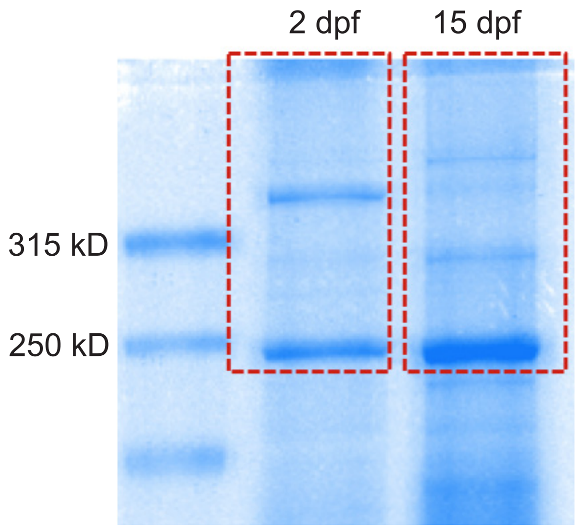

Figure S7.

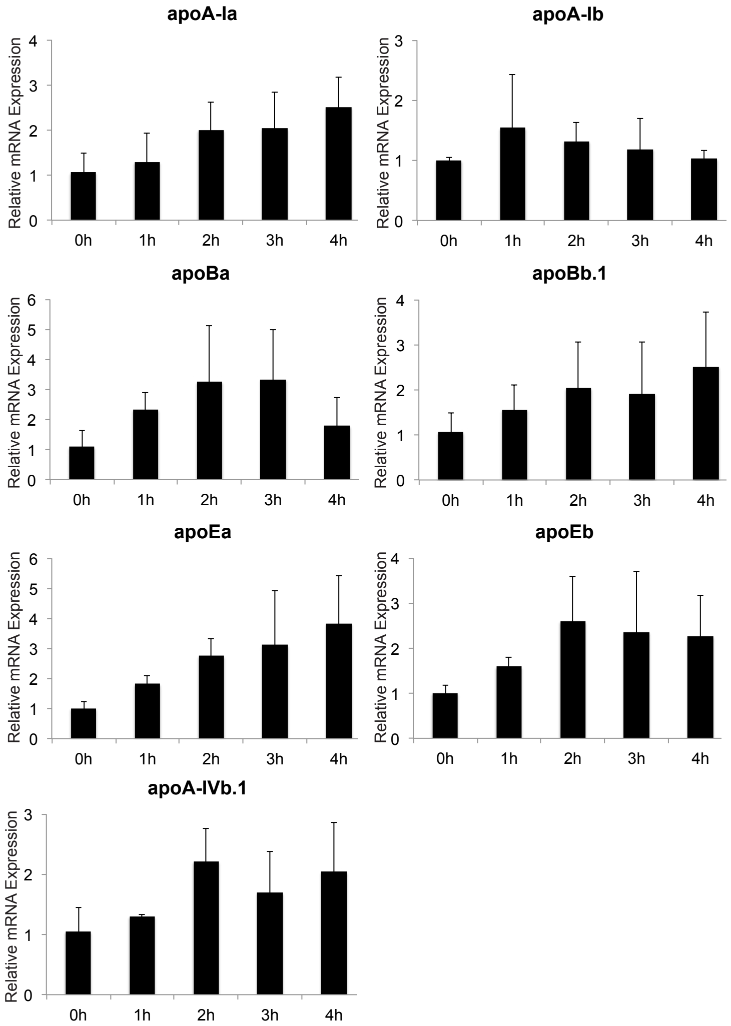
apoA-Ia
apoA-Ib
apoBa
apoBb.1
apoEa
apoEb
apoA-IVb.1
Relative mRNA Expression
0h
1h
2h
3h
4h

Figure S8.

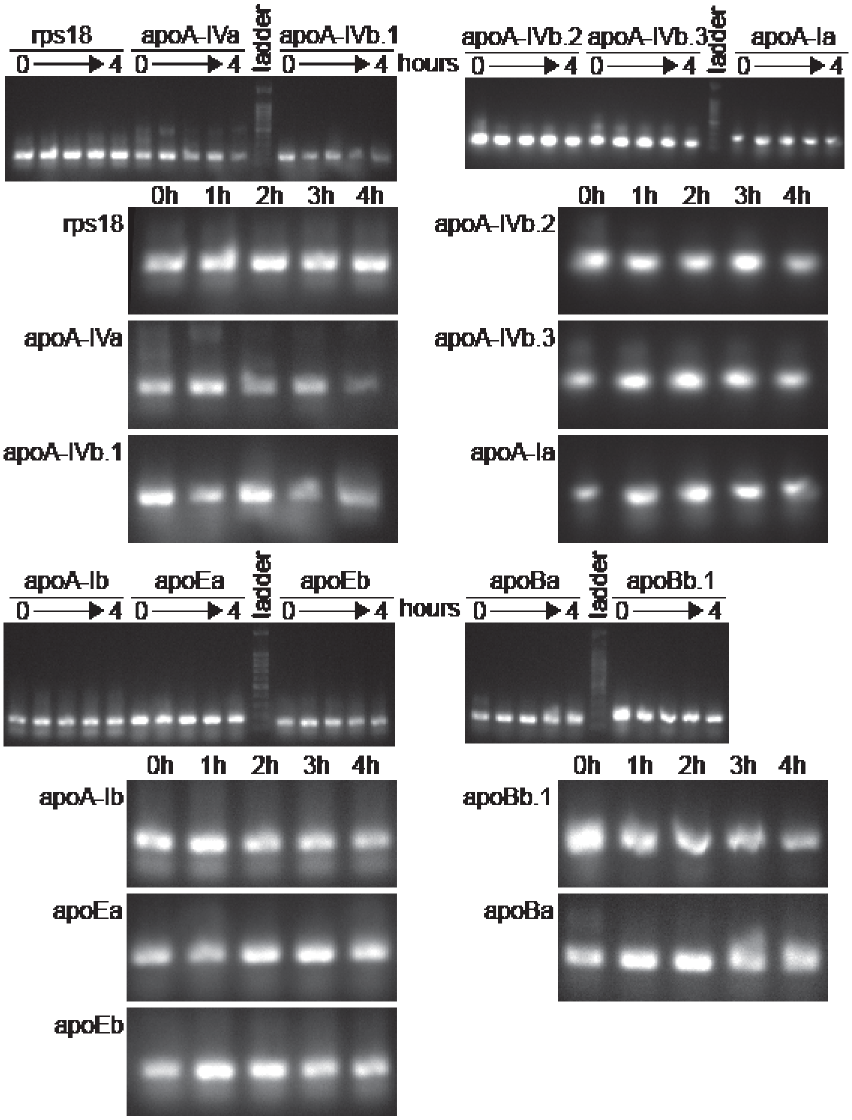
rps18
apoA-IVa
ladder
apoA-IVb.1
0
4
hours
apoA-IVb.2
apoA-IVb.3
apoA-Ia
0h
1h
2h
3h
4h
apoA-Ib
apoEa
apoEb
apoBa
apoBb.1

Figure S9.

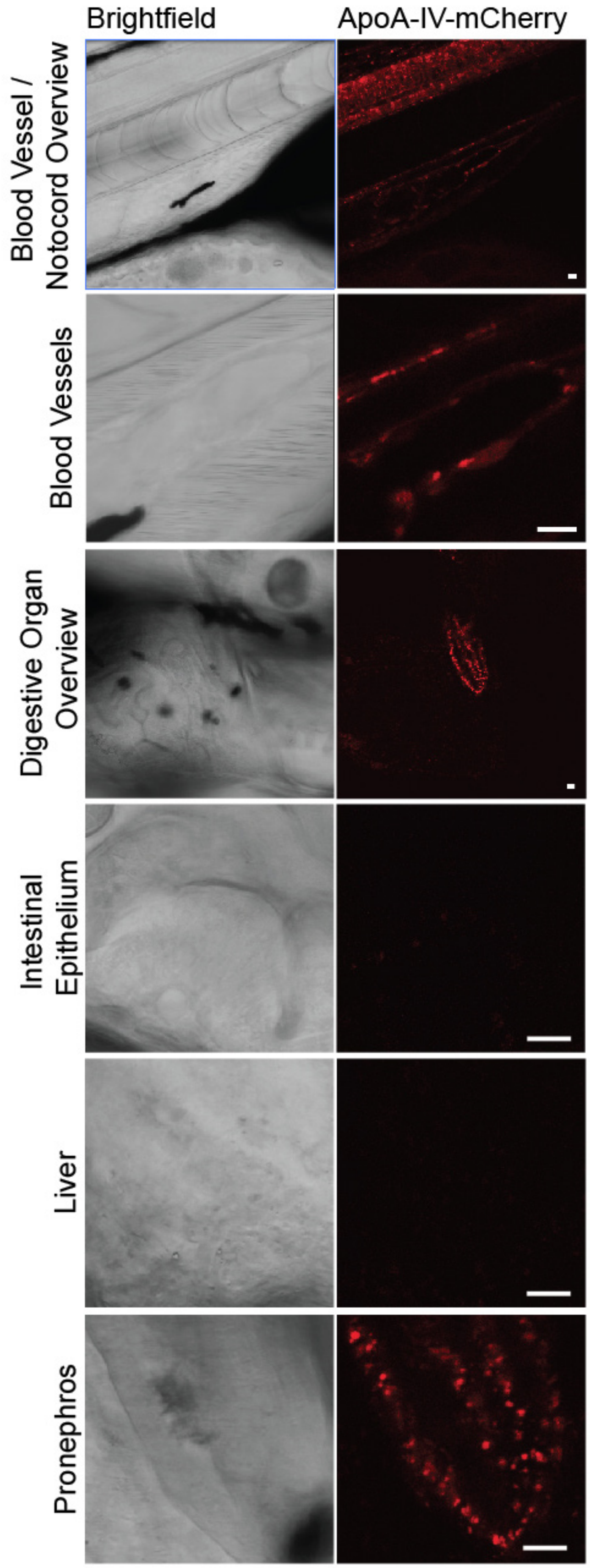
Brightfield
ApoA-IV-mCherry
Blood Vessel / Notocord Overview
Blood Vessels
Digestive Organ Overview
Intestinal Epithelium
Liver
Pronephros

Table S1. Accession numbers of zebrafish and human apolipoproteins genes.

Gene	Species	Accession Number(s)	Historic Name	Chromosome
apoA-Ia	*Danio rerio*	ENSDARG00000012076	n/a	5
apoA-Ib	*Danio rerio*	ENSDARG00000086583	n/a	15
apoBa	*Danio rerio*	ENSDARG00000042780 (for LC-MS/MS: Q5TZ29)	*apoB (2)*	17
apoBb.1	*Danio rerio*	ENSDARG00000022767, (for LC-MS/MS: E7FBD3)	*apoBb*	20
apoBb.2	*Danio rerio*	ENSDARG00000075016, (for LC-MS/MS: F1QMP5)	*apoB like*	20
apoEa	*Danio rerio*	ENSDARG00000086370	n/a	19
apoEb	*Danio rerio*	ENSDARG00000040295	n/a	6
apoA-IVa	*Danio rerio*	ENSDARG00000086281	*apoA-IV (3)*	19
apoA-IVb.1	*Danio rerio*	ENSDARG00000049298	*apoA-IV*	16
apoA-IVb.2	*Danio rerio*	ENSDARG00000020866	*apoA-IV like*	16
apoA-IVb.3	*Danio rerio*	ENSDARG00000094929	*apoA-IV (4)*	16
APOA-I	*Homo sapiens*	NP_000030.1	n/a	11
APOB	*Homo sapiens*	NP_000375.2	n/a	2
APOE	*Homo sapiens*	NP_000473.2	n/a	19
APOA-IV	*Homo sapiens*	NP_000032.1	n/a	11

Table S2. Primers used to synthesize in situ hybridization probes.

Gene	Primers
ApoA-Ia	F: TCG TGG CTC TTG CAC TGA C<br>R: TCC ATG TAG GGC TCC ATG C
ApoA-Ib	F: CTC GCC CTC ACC GTA TTC CT<br>R: CGA ACT TCT GGA GGG CCT TG
ApoBa	F: TCA GTT TCC CGT CCC TCA C<br>R: AAG ATC ATC CTG GGG AAA GC
ApoBb.1	F: GTC GTT ATG CTT TTG CAC CA<br>R: CGA TGG CAT TGT CAA TCA AG
ApoBb.2	F: TCC ATG CCA ATC TCA ATC AAG TC<br>R: AGT CCA ACA GGC TGC CAT AC
ApoEa	F: GAA CGA CTG CGA TCC AAG C<br>R: GAG CTC CTG CAT TCG TGA GT
ApoEb	F: ACG GCA TGG TGC AAA ACA TC<br>R: TGT CCA GTA AAG GAC AGT GC
ApoA-IVa	F: GAA CGA GTC ACA CGG CAT GT<br>R: CCA GCT TTG CCT TCA GCT CT
ApoA-IVb.1	F: GTC TTA CAC CCT ATG CTG AAG AC<br>R: GAACAA AAC CAA TCC CCA ATT CC
ApoA-IVb.2	F: AGG AGT TCC AGA AGA CCG TGA C<br>R: GCA GCA GTT TTC AAA AGT CTG TTG G
ApoA-IVb.3	F: ACA GAC TAA AAG TGA AGA CCA TGA AG<br>R: G GAT CAA ATA TGT CAA ATT TCG AAG TTT GTG

Table S3. Real-time PCR primers used in this study.

Gene	Primers
ApoA-Ia	F: CCA ATT TGT TCC AGG CTG AT<br>R: CAA CTG GGT GGA GAT GGT CT
ApoA-Ib	F: GCC CTA CGT CCA GGA GTA CA<br>R: TTA CTC CTT GCT GGC GAA CT
ApoBa	F: TGA CCT CAA GCA CGT CAC TC<br>R: GGG GAA AAC CAG CAC TTG TA
ApoBb.1	F: GCT TGA AGG AAC CAG CAG TC<br>R: AGT TGG TGG TTG GCA TTA GC
ApoEa	F: GCA GAA CTC TGA TGA GCT CAA GAA CAA AGC<br>R: AAG TAA GGC TCC AGA CGA TCC TTC ACA TCT
ApoEb	F: CTC TTG TGG TAT TCT TTG CTC TGG CAG TTT<br>R: TTG CAC CAT GCC GTC AGT TTG TGT GTT GAG
ApoA-IVa	F: GAC CCA GCT CAA GCC TTA TG<br>R: GAC CCA GCT CAA GCC TTA TG
ApoA-IVb.1	F: GAG TTC CAG AAA ACT GTG AGT CCT CTA GCT<br>R: TCG TAC AGA GAG ATC AGC TGG TCT TTT AGG
ApoA-IVb.2	F: TTG TGG TCT TTG CAC TTG CT<br>R: TCA TCT TGA CGG TTT CCT CTG
ApoA-IVb.3	F: TGA AGG TTC TTG TGG TGC TC<br>R: AAT GGA TTC CTC TGC GGT TT
18S	F: TGC AGA ACC CTC GCC AGT ACA AAA TCC CAG<br>R: CCA GAA GTG ACG GAG ACC ACG GTG AGC CCT
